# Chain-Selective Isotopic Labeling of the Heterodimeric Type III Secretion Chaperone, Scc4:Scc1, Reveals the Total Structural Rearrangement of the *Chlamydia trachomatis* Bi-Functional Protein, Scc4

**DOI:** 10.3390/biom10111480

**Published:** 2020-10-24

**Authors:** Thilini O. Ukwaththage, Samantha M. Keane, Li Shen, Megan A. Macnaughtan

**Affiliations:** 1 Department of Chemistry, Louisiana State University, Baton Rouge, LA 70803, USA; tukwat1@lsu.edu (T.O.U.); skeane1@lsu.edu (S.M.K.); 2 Department of Microbiology, Immunology, and Parasitology, Louisiana State University Health Sciences Center, New Orleans, LA 70112, USA; lshen@lsuhsc.edu

**Keywords:** type III secretion, chaperone, transcription, *Chlamydia trachomatis*, NMR, Scc4, Scc1, selective isotopic labeling

## Abstract

Scc4 is an unusual bi-functional protein from *Chlamydia trachomatis* (*CT*) that functions as a type III secretion system (T3SS) chaperone and an RNA polymerase (RNAP)-binding protein. Both functions require interactions with protein partners during specific stages of the *CT* developmental cycle. As a T3SS chaperone, Scc4 binds Scc1 during the late stage of development to form a heterodimer complex, which chaperones the essential virulence effector, CopN. During the early-middle stage of development, Scc4 regulates T3SS gene expression by binding the σ^66^-containing RNAP holoenzyme. In order to study the structure and association mechanism of the Scc4:Scc1 T3SS chaperone complex using nuclear magnetic resonance (NMR) spectroscopy, we developed an approach to selectively label each chain of the Scc4:Scc1 complex with the ^15^N-isotope. The approach allowed one protein to be visible in the NMR spectrum at a time, which greatly reduced resonance overlap and permitted comparison of the backbone structures of free and bound Scc4. ^1^H,^15^N-heteronuclear single quantum coherence spectra of the ^15^N-Scc4:Scc1 and Scc4:^15^N-Scc1 complexes showed a total structural rearrangement of Scc4 upon binding Scc1 and a dynamic region isolated to Scc1, respectively. Development of the chain-selective labeling approach revealed that the association of Scc4 and Scc1 requires partial denaturation of Scc1 to form the high affinity complex, while low affinity interactions occurred between the isolated proteins under non-denaturing conditions. These results provide new models for Scc4′s functional switching mechanism and Scc4:Scc1 association in *CT*.

## 1. Introduction

*Chlamydia trachomatis (CT)* is a gram-negative, obligate intracellular bacterial pathogen with distinct biovars that cause the most common sexually transmitted bacterial diseases or infectious blindness due to trachoma, a neglected infectious disease [1,2]. *CT* utilizes a type three secretion system (T3SS) to establish and maintain infection by delivering over 100 effector proteins into the host epithelial cell [3,4,5,6]. Once the bacteria enter the host cell, they localize in a specialized membrane-surrounded vacuole, termed an inclusion, where the effector proteins defend against the host’s immune response and provide critical nutrients from the host cell. *CT* undergoes a unique, biphasic developmental cycle within the inclusions from infectious elemental bodies (EBs) to noninfectious, vegetative reticulate bodies (RBs). RBs transit back to EBs prior to release of EBs from the host cell to reinitiate new infections. The T3SS and transcriptional control of the developmental cycle are essential for infection, and Scc4 (specific chlamydia chaperone 4, formerly CT663, UniprotKB O84670) is a bi-functional protein with implications to both systems.

**Figure 1:**
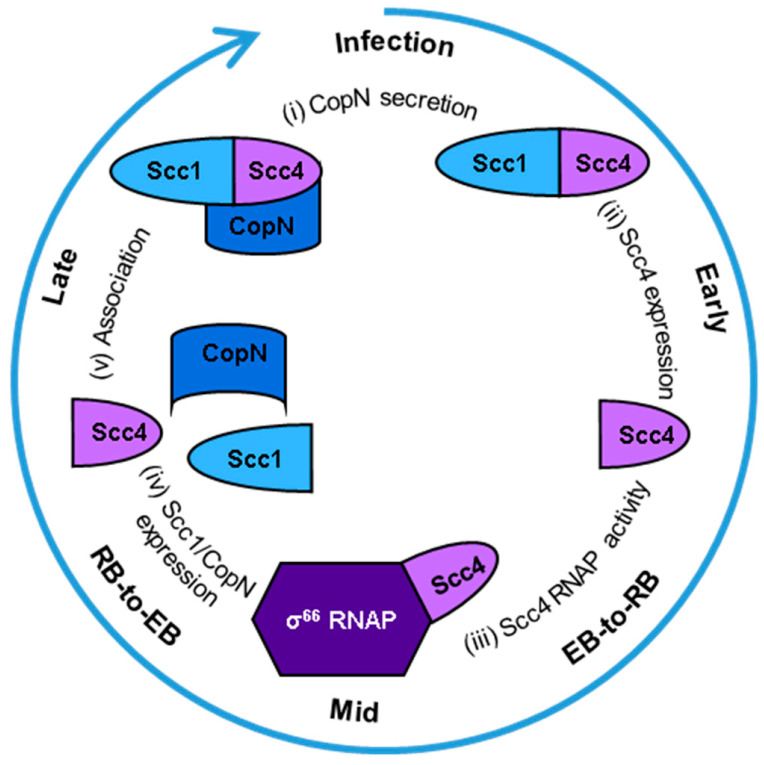
Model of Scc4′s protein-protein interactions during the *Chlamydia trachomatis* developmental cycle. Starting at the top of the circle and going clockwise: (**i**) Infection is initiated when the T3SS needle on an EB contacts a host cell. The T3SS chaperone complex of Scc4:Scc1 delivers CopN to be secreted into the host’s cytosol, and the complex remains in the bacterial cytosol. (**ii**) Scc4 is expressed in the early stage of the developmental cycle and (**iii**) functions as a σ^66^-RNAP binding protein during the EB-to-RB transition in the middle of the developmental cycle. (**iv**) Scc1 and CopN are expressed during the RB-to-EB transition, late in the cycle. (**v**) Scc4 associates with Scc1, and the complex binds CopN in the EBs. The EBs are released from the host cell to start a new cycle of infection.

Scc4 switches between two functions that depend on different protein-protein interactions at specific stages of the developmental cycle with impacts on chlamydial development, CopN secretion, and selective gene expression [7]. A model of these protein-protein interactions is shown in Figure 1. In extracellular EBs, a T3SS is poised for infection. Scc4 binds Scc1 (specific chlamydia chaperone 1 or CT088, UniprotKB O84090) to function as a T3SS chaperone for the exported virulence effector, *Chlamydia* outer protein N (CopN) [8,9,10]. CopN is proposed to be the plug protein of the T3SS and the first virulence factor secreted into the host cell [11,12]. Infection (Figure 1, (i)) is initiated when EBs contact the host cell and the T3SS is activated, delivering CopN to the host’s cytosol and leaving the Scc4:Scc1 complex in the bacterial cytosol. Within the host cell, CopN targets cytoskeletal structures, tubulin or microtubules, to arrest the cell cycle [13,14]. The fate of the Scc4:Scc1 complex after secretion of CopN is unknown. During the early and middle stages, Scc4 is expressed [15,16] (Figure 1, (ii)) and begins its function as an RNAP-binding protein (Figure 1, (iii)) to promote EB-to-RB differentiation and RB replication. Scc4 regulates *CT*’s σ^66^-dependent transcription by directly interacting with σ^66^ region 4 and the flap tip domain of the β subunit of RNA polymerase (RNAP) [15]. Overexpression of Scc4 in *CT* accelerates the development of the inclusions by regulating T3SS gene expression [7]. During the middle to late stages of the cycle, Scc1 and CopN are expressed [16,17] (Figure 1, (iv)) and RBs redifferentiate to EBs. Scc4 associates with Scc1 (Figure 1, (v)) to chaperone CopN until a new cycle of infection starts. CopN does not associate with either Scc4 nor Scc1 until the Scc4:Scc1 complex is formed [16]. Our current model is Scc4 and Scc1 associate in *CT* when Scc4 is abundant and Scc1 is newly expressed followed by association with newly expressed CopN.

In the present study, an approach to produce chain-selectively labeled Scc4:Scc1 complexes was developed and used to compare the 3-dimensional backbone structure and dynamics of free and Scc1-bound Scc4 and to study the mechanisms of Scc4 and Scc1 association. The NMR resonance assignments (^1^H, ^13^C, and ^15^N) of Scc4 revealed a dynamic protein [18], but the investigation of the Scc4:Scc1 complex using NMR is more difficult due to its larger size (33.5 kDa) and resonance overlapping. For the structural studies of protein complexes, isotopic labelling of selected segments or chains of a protein greatly reduces the overlap [19,20]. We tested several methods to produce the chain-selectively labeled Scc4:Scc1 complex, where only one protein is isotopically labeled and visible in the NMR spectrum at a time. Through these tests, we reveal new information about the functional switching mechanism of Scc4, association of Scc4 and Scc1, and the total backbone structural rearrangement of Scc4 upon binding Scc1.

## 2. Materials and Methods

### 2.1. Materials

The protein expressions were conducted using the previously reported recombinant expression vectors of pACYCHis_6_-Scc1, pET28Scc4, and pCDFScc4 [16]. The proteins that are encoded in these vectors correspond to Scc1 with an *N*-terminal His_6_-tag (His_6_-Scc1), Scc4, and Scc4 with an *N*-terminal His_6_-tag (His_6_-Scc4), respectively. The plasmid pScc1-FT [21] was generously gifted by Dr. Ken Fields (University of Kentucky, College of Medicine, Lexington, KY, USA), which encodes Scc1 with a *C*-terminal FLAG tag (Scc1-FLAG) [22]. Millipore Direct-Q 3 ultrapure water system was used as the water source.

### 2.2. General Protocols

Centrifugal filters with a molecular weight cutoff of 10 kDa were used to exchange protein sample buffer and concentrate samples prior to analysis. For exchanging the buffer, the samples were concentrated to 0.25–0.5 mL and diluted to 4 mL at least 4 times. Purified protein samples were stored at −20 °C and concentrated to 0.35–0.5 mM immediately before NMR analysis. The Pierce Coomassie (Bradford) Plus protein assay kit was used to determine protein concentrations following the manufacturers 96-well plate procedure. Sodium dodecyl sulfate polyacrylamide gel electrophoresis (SDS-PAGE) analysis was performed by mixing equal volumes of the samples with 2X Laemmli buffer with 5% β-mercaptoethanol (*v*/*v*), heating at 85−95 °C for 3 min, and running on a 4–20% Tris/glycine gel at 120 V for 60 min in running buffer (25 mM tris (hydroxymethyl) aminomethane hydrochloride (Tris), 192 mM glycine, 0.1% SDS, pH 8.3). Native gel analysis was performed by mixing the sample with equal volumes of 2X native sample buffer (62.5 mM Tris, 40% glycerol, 0.01% bromophenol blue (*w*/*v*)) and running on a 4–20% Tris/glycine gel at 120 V for 60 min in running buffer (25 mM Tris, 192 mM glycine, pH 8.3). All gels were stained in brilliant blue R-250 solution (1.0 g/L brilliant blue R-250 dye, 50% methanol, 40% water, and 10% acetic acid) for 1 h. After de-staining in 50% water, 40% methanol, 10% acetic acid, the gels were imaged using a Bio-Rad Gel Doc EZ System. Nickel immobilized affinity chromatography (Ni-IMAC) was performed using a fast protein liquid chromatography system, 1–4 mL of nickel-charged resin in a 1 cm inner diameter column, absorbance detection at 280 nm, and a fraction collector. The flow rate was 1 mL/min for sample loading and 2 mL/min for wash and elution steps. Wash steps were performed using 20 mL of Tris or phosphate buffer. Tris buffer is 20 mM Tris, 300 mM NaCl, 2 mM imidazole, and 5% (*w*/*v*) glycerol at pH 8.0. Phosphate buffer is 50 mM sodium phosphate, 300 mM NaCl, 2 mM imidazole, and 5% glycerol (*w*/*v*) at pH 8.0. Elution steps were performed with 10 mL of 0.5% sarkosyl (sodium lauryl sarcosinate: a non-denaturing detergent) in Tris buffer or 500 mM imidazole in Tris or phosphate buffer. Qualitative size exclusion chromatography (SEC) was performed using a fast protein liquid chromatography system with a ProteoSEC 16/60 3-70 HR column, NMR buffer, 2 mL/min flow rate, absorbance detection at 280 nm, and a fraction collector. NMR buffer is 50 mM sodium phosphate, 10 mM of dithiothreitol (DTT), pH 7.3.

### 2.3. Protein Expression and Purification

The in vivo-associated complexes, Scc4:His_6_-Scc1 and His_6_-Scc4:Scc1-FLAG, were expressed and purified as described by Ukwaththage, et al. [22]. Briefly, Scc4 and His_6_-Scc1 were co-expressed from the pET28Scc4 and pACYCHis_6_-Scc1 vectors in *E. coli* BL21-Gold (DE3) cells, and His_6_-Scc4 and Scc1-FLAG were co-expressed from the pCDFScc4 and pScc1-FT vectors in *E. coli* T7 Express cells. The cultures were first grown in LB medium to an optical density at 600 nm of 0.6, and the cells were transferred to M9 minimal medium (with ammonium chloride or ^15^N-ammonium chloride). The protein expression was induced with 0.5 mM isopropyl β-d-1-thiogalactopyranoside (IPTG), and the cultures were grown at 16 °C with 250 rpm shaking for 16 h. The cells were harvested by centrifugation (4000× *g*, 15 min, 4 °C) and lysed using a French pressure cell 3 times. The protein complexes were purified from the clarified lysate using Ni-IMAC with 4 mL of resin and Tris buffer followed by SEC with NMR buffer. The purified proteins were analyzed using SDS-PAGE and the Coomassie protein assay.

Co-expression cultures of the Scc4:His_6_-Scc1 and His_6_-Scc4:Scc1-FLAG complexes were used to purify Scc4 and Scc1-FLAG and prepare immobilized His_6_-Scc1 as described by Ukwaththage, et al. [22]. Briefly, the complexes were immobilized on 4 mL of Ni-IMAC resin, and 0.5% sarkosyl in Tris buffer was used to dissociate the complexes and elute either Scc4 or Scc1-FLAG, leaving the His-tagged proteins (His_6_-Scc1 or His_6_-Scc4) on the resin. The immobilized His_6_-Scc1 was washed with Tris buffer to remove residual sarkosyl. Sarkosyl was removed from the purified Scc4 and Scc1-FLAG proteins by diluting the samples 5-fold with NMR buffer and exchanging the buffer to NMR buffer using centrifugal filters. The purified proteins and the immobilized His_6_-Scc1 were analyzed using SDS-PAGE, and the purified Scc4 and Scc1-FLAG proteins were analyzed using the Commassie protein assay.

Scc4 and His_6_-Scc1 were expressed individually in *E. coli* BL21-Gold (DE3) cells using the same method as the expression of the Scc4:His_6_-Scc1 and His_6_-Scc4:Scc1-FLAG complexes. Scc4 in clarified lysate and His_6_-Scc1 inclusion bodies were prepared by suspending the harvested cells in 20 mL of Tris buffer with 2 Pierce EDTA-free protease inhibitor mini tablets, 2.3 mL of 10X BugBuster protein extraction reagent, and 1 μL of Benzonase nuclease and lysing with a French press (SLM Instruments Inc./American Instrument Co., Urbana, IL, USA) 3 times. The Scc4 cell lysate was clarified by centrifugation (25,000× *g* for 20 min at 4 °C). His_6_-Scc1 inclusion bodies were purified by washing the lysed pellet with 20 mL each of 1× BugBuster in Tris buffer, 1X BugBuster in Tris buffer with 200 μg/mL of hen egg white lysozyme, and 1X BugBuster in Tris buffer using centrifugation at 15,000× *g* for 20 min at 4 °C to remove the supernatant between each step. The purified His_6_-Scc1 inclusion bodies were centrifuged at 25,000× *g* for 20 min at 4 °C to remove residual supernatant and analyzed using SDS-PAGE.

### 2.4. NMR Analysis

Protein samples were prepared in NMR buffer using SEC and centrifugal filters to a final concentration of 0.35–0.5 mM. The samples were transferred to 5 mm D_2_O-susceptibility matched Shigemi tubes, and 5 µM sodium 3-(trimethylsilyl)-1-propanesulfonate (DSS) and 10% D_2_O were added for chemical shift referencing and locking, respectively. ^1^H,^15^N-Heteronuclear single quantum coherence (HSQC) spectra were recorded on an Advance III HD 500 MHz instrument (Bruker Corp., Billerica, MA, USA) at 300 K (27 °C) with 32–64 scans and 256 increments. Spectra were processed and analyzed with Bruker Topspin software.

For the chemical shift perturbation analysis, ^15^N-Scc4 was concentrated to 0.5 mM and transferred to a 5 mm D_2_O-susceptibility matched Shigemi tube. DSS (5 µM) and 10% D_2_O were added for chemical shift referencing and locking, respectively. Scc1-FLAG was concentrated to make two stock solutions in NMR buffer: Stock 1 was 3.75 mM and Stock 2 was 7.5 mM. ^1^H,^15^N-HSCQ spectra were collected on an Advance III HD 500 MHz instrument (Bruker Corp., Billerica, MA, USA) at 300 K (27 °C) with 8 scans and 256 increments. The ^15^N-Scc4 sample was analyzed with increasing mole fractions of Scc1-FLAG (0, 0.25, 0.5, 1.0, 1.5, and 2.0 moles of Scc1-FLAG to moles of ^15^N-Scc4). Stock 1 was used for the 0.25 and 0.5 mole fraction additions of Scc1-FLAG, and Stock 2 was used to increase the mole fraction to 1.0, 1.5, and 2.0. The sample volume over the titration was 300–350 μL, which is within the detectable region of the NMR probe such that dilution did not affect the signal intensities. After each addition of Scc1-FLAG, the samples were incubated at room temperature for 30 min. For the 1:2 molar ratio (^15^N-Scc4:Scc1-FLAG) sample, an additional HSQC spectrum was collected after the sample was incubated overnight at 4 °C. The chemical shift perturbation was calculated by adding the combined chemical shift change [23] using a ^15^N shift scaling factor of 0.14 for each step of the titration (since the changes were non-linear).

### 2.5. On-Column Association of ^15^N-Scc4 and His_6_-Scc1

His_6_-Scc1 immobilized on Ni-IMAC resin and samples of ^15^N-Scc4 (purified or in clarified lysate) were mixed to test complex formation. Purified ^15^N-Scc4 or ^15^N-Scc4 in clarified lysate were each loaded onto the Ni-IMAC resin with immobilized His_6_-Scc1. The sample that flowed through the column was collected for SDS-PAGE analysis. The resin was washed with Tris buffer, and the proteins were eluted with 500 mM imidazole in Tris buffer. SDS-PAGE analysis was used to determine if ^15^N-Scc4 associated with His_6_-Scc1 and eluted with imidazole or if ^15^N-Scc4 failed to associate and flowed through the column. The on-column associated complex from mixing ^15^N-Scc4 in clarified lysate with immobilized His_6_-Scc1 was tested for stability over 2 days at room temperature and compared to the stability of the in vivo-associated complex using native PAGE and qualitative SEC analyses. The differences in the qualitative SEC elution times of the in vivo- and on-column-associated complexes should not be interpreted quantitatively to measure molecular weight; because, while the 0 and 2 d complexes were analyzed 2 days apart and can be compared to assess stability of the complex, the different complexes were analyzed weeks to months apart and the drift in the pump flow rates and column condition were not corrected by calibration.

### 2.6. Renatured-Association of Scc4 and His_6_-Scc1 with Chain Selective Labeling

Renatured-association of Scc4 and His_6_-Scc1 involved diluting denatured His_6_-Scc1 with refolding buffer and adding it dropwise to clarified Scc4 lysate. Four different refolding buffers were tested. The standard refolding buffer (Buffer 4) is 125 mM Tris, 175 mM NaCl, 10 mM DTT, and pH 7.7. Additives to the standard buffer are 5 mM glutathione (GSH), 0.5 mM oxidized glutathione (GSSH), and 5 M L-arginine (Buffer 1), 5 mM GSH and 0.5 mM GSSH (Buffer 2), and 5 M L-arginine (Buffer 3). Purified His_6_-Scc1 inclusion bodies from a 500 mL culture were denatured in 8 mL of 6 M guanidine hydrochloride (GuHCl) in Buffer 4 by vortexing the solution until it became clear. Samples of the denatured His_6_-Scc1 (1 mL) were diluted by dropwise addition of 2 mL of each refolding buffer, reducing the GuHCl concentration to 2 M. Clarified Scc4 lysate (23 mL) from a 1 L culture was split into 4 aliquots of 5 mL. The diluted His_6_-Scc1 samples were added dropwise to the clarified lysate resulting in a final GuHCl concentration of 0.75 M. Each mixture was purified by Ni-IMAC using 1 mL of resin and Tris buffer. The purified proteins were analyzed using SDS-PAGE and the Coomassie protein assay.

Chain selectively labeled Scc4:His_6_-Scc1 complexes (^15^N-Scc4:His_6_-Scc1 and Scc4:^15^N-His_6_-Scc1) were prepared using the renaturing method described above with Buffer 4. Denatured His_6_-Scc1 (or ^15^N-His_6_-Scc1) from a 500 mL culture (8 mL) was diluted with 16 mL of Buffer 4 and added dropwise with vortexing to 23 mL of clarified ^15^N-Scc4 (or Scc4) lysate from a 1 L culture (1.0 M final GuHCl concentration). The protein mixtures were incubated for 10 min at room temperature and centrifuged at 8000× g for 10 min at 4 °C to remove any precipitated proteins. The clarified protein mixtures were purified by Ni-IMAC using 3 mL of resin and phosphate buffer followed by SEC with NMR buffer. The purified proteins were analyzed using SDS-PAGE and the Coomassie protein assay. The renatured complex was tested for stability over 2 days at room temperature and compared to the stability of the in vivo-associated complex using native PAGE and qualitative SEC analyses. The differences in the qualitative SEC elution times of the in vivo-associated and renatured complexes should not be interpreted quantitatively (refer to Section 2.5 for details).

## 3. Results and Discussion

### 3.1. Crowded NMR Spectra of In Vivo-Associated, ^15^N-labeled Scc4:Scc1 Complexes

In our previous work, we showed that Scc4 and Scc1 associate in vivo when co-expressed in *E. coli* [16]. The interaction was strong allowing for the complex to be purified by Ni-IMAC when either Scc4 or Scc1 had a His_6_-tag. Two variations of the complex (different by their affinity purification tags) were produced for crystallization trials, Scc4:His_6_-Scc1 and His_6_-Scc4:Scc1-FLAG, with no crystals. As an alternative to X-ray crystallography for protein structure determination, the suitability of the complexes for NMR structure determination was investigated. To this end, ^15^N-labeled Scc4:His_6_-Scc1 and His_6_-Scc4:Scc1-FLAG complexes were produced by metabolic labeling in *E. coli* using ^15^N-supplemented minimal medium. The yields of the complexes from the minimal media cultures were similar to the yields from LB media cultures [22] with 10–12 mg per L culture for Scc4:His_6_-Scc1 and 18–20 mg per L culture for His_6_-Scc4:Scc1-FLAG. As expected, the purified complexes showed a 1:1 molar ratio on an SDS-PAGE gel and eluted as a single peak in SEC, indicating the successful purification of the in vivo-associated complexes (Figures S1 and S2).

**Figure 2:**
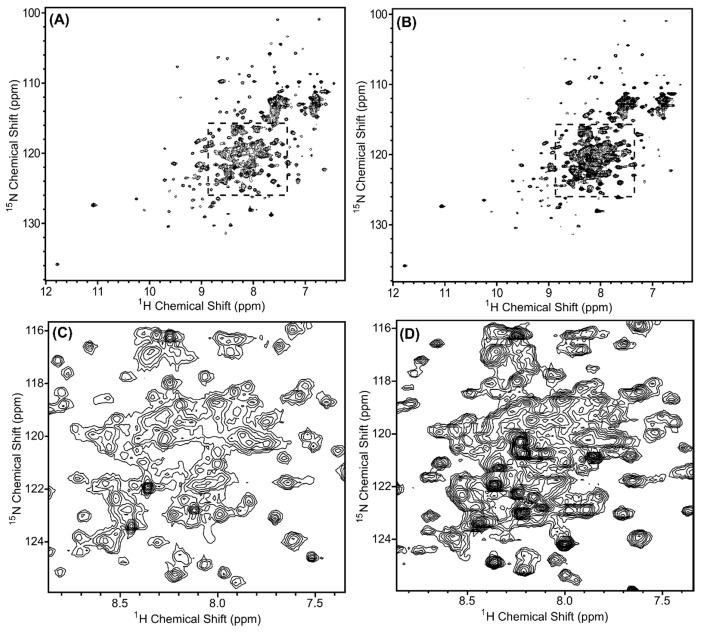
^1^H-^15^N HSQC spectra of the in vivo-associated, ^15^N-labeled protein complexes (**A**,**C**) Scc4:His_6_-Scc1 and (**B**,**D**) His_6_-Scc4:Scc1-FLAG. The regions shown in (**C**,**D**) are expanded from the dashed boxes shown in (**A**,**B**). The complexes were 0.35 mM concentration in 50 mM sodium phosphate, 10 mM of DTT, pH 7.3 buffer, and the spectra were collected with 32 scans using a Bruker AVIII 500 MHz spectrometer. Overlays of the spectra are shown in Figure S3.

In order to determine the structure of a protein or protein complex using NMR spectroscopy, the first step is to evaluate the quality of the ^1^H, ^15^N-HSQC spectrum of the sample. In this spectrum, we expect a peak for each amide group (except proline), which includes the amides along the backbone and in the sidechains. If the protein is folded, we expect the peaks to be of similar intensity (similar peak widths) and dispersed. The ^1^H, ^15^N-HSQC spectra of ^15^N-labeled Scc4:His_6_-Scc1 and His_6_-Scc4:Scc1-FLAG are shown in Figure 2A,B, respectively, and overlaid in Figure S3. As expected, the peak positions in each spectrum are the same, indicating that the two complexes have the same fold, with the exception of a subset of peaks likely belonging to the different purification tags. The Scc4:His_6_-Scc1 spectrum has better NMR spectral properties compared to the His_6_-Scc4:Scc1-FLAG spectrum. The relative intensity of the peaks for the Scc4:His_6_-Scc1 complex (Figure 2A) is more uniform than the His_6_-Scc4:Scc1-FLAG complex (Figure 2B), and the Scc4:His_6_-Scc1 complex has fewer peaks near the center of the spectrum (Figure 2C,D). We have observed similar differences between ^1^H,^15^N-HSQC spectra of Scc4 and His_6_-Scc4, where the His_6_-tag results in poorer quality spectra [22]. Similarly, the Scc1 purification tags, the *N*-terminal His_6_-tag and the *C*-terminal FLAG tag, may contribute to differences in the spectra. In the Scc4:His_6_-Scc1 spectrum, 298 peaks (out of an expected 364 peaks) were counted giving 82% of the expected amide peaks (including sidechain peaks). While this percentage is an excellent result for a relatively large complex (33.5 kDa), the large number of peaks and crowded region in the center of the spectrum prevents unambiguous peak assignment, which is necessary for structure determination. An alternative approach to using the fully ^15^N-labeled complex is to selectively label each chain in the complex such that one protein is visible in the NMR spectrum at a time reducing resonance overlap.

### 3.2. On-Column-Associated Scc4:Scc1 Is Less Stable Than the In Vivo-Associated Complex

Chain-selective labeling of a protein:protein complex is a great way to simplify the NMR spectra and increase the assignment coverage of the proteins. It is also useful for distinguishing between intra- and intermolecular nuclear Overhauser effects (used to measure distance restraints), which are needed to determine the quaternary structure of the complex. Regardless of the method used to make a chain-selectively labeled complex, the two proteins must interact under conditions that allow for stable association into the native quaternary structure.

**Figure 3:**
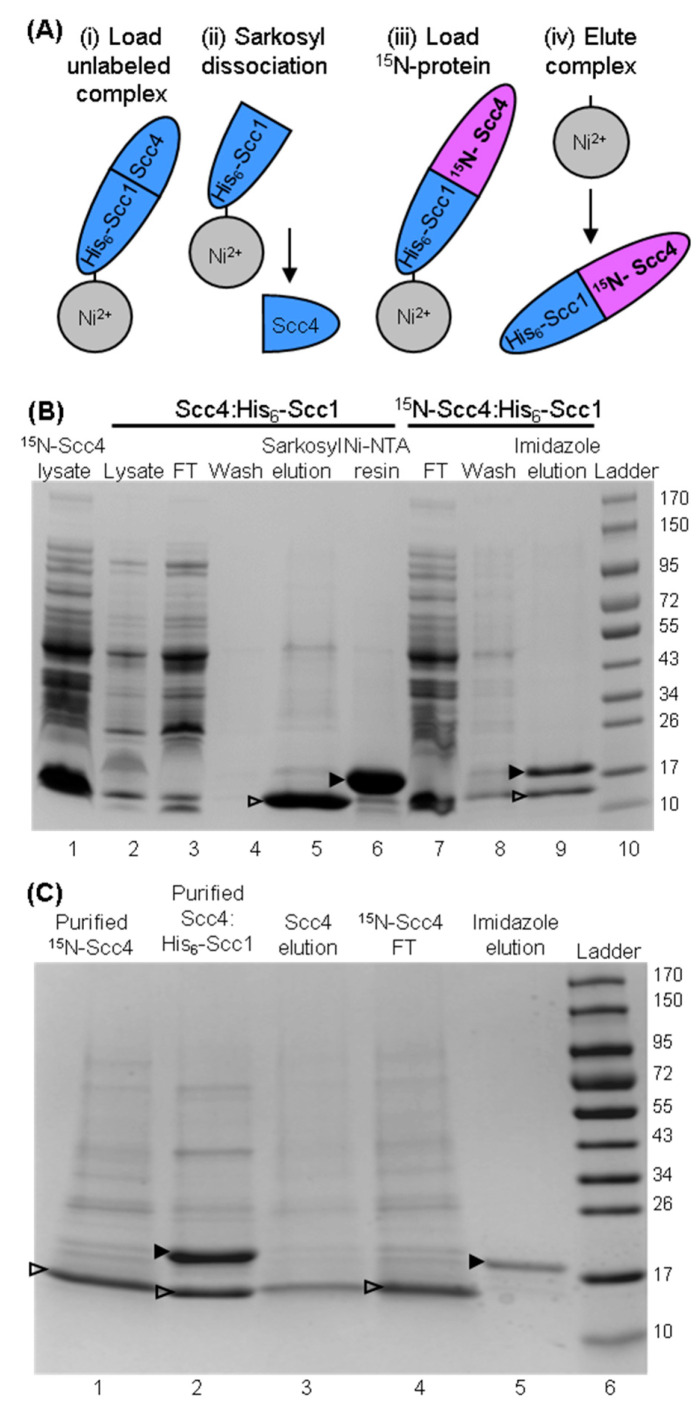
Preparation of chain-selectively labeled complex using on-column association with ^15^N-Scc4 in clarified lysate or purified ^15^N-Scc4. (**A**) Scheme for making chain-selectively labeled complex on-column. The unlabeled, in vivo-associated complex is (**i**) loaded onto Ni-IMAC resin and washed. (**ii**) Sarkosyl buffer is used to dissociate the complex resulting in the elution of unlabeled Scc4. Wash buffer is used to remove residual sarkosyl from the immobilized His_6_-Scc1. (**iii**) ^15^N-labeled Scc4 is loaded and associates with His_6_-Scc1 on the column. The complex is washed to remove excess ^15^N-Scc4, and (**iv**) the chain-selectively labeled complex is eluted with imidazole buffer. The Scc4:^15^N-His_6_-Scc1 complex is made by switching the labeled components, i.e., ^15^N-labeled complex in (**i**) and unlabeled Scc4 in (**iii**). (**B**) SDS-PAGE analysis of the on-column association of immobilized His_6_-Scc1 and ^15^N-Scc4 in clarified lysate. Clarified lysate samples containing ^15^N-Scc4 and unlabeled, co-expressed Scc4:His_6_-Scc1 complex are shown in lanes 1 and lane 2, respectively. The immobilization of His_6_-Scc1 onto Ni-IMAC resin is shown in lanes 3–6 with samples of the Scc4:His_6_-Scc1 lysate that flowed through the resin (lane 3), wash (lane 4), sarkosyl elution of Scc4 from the immobilized Scc4:His_6_-Scc1 complex (lane 5), and Ni-IMAC resin with immobilized His_6_-Scc1. On-column association of ^15^N-Scc4 with the immobilized His_6_-Scc1 is shown in lanes 7-9 with the ^15^N-Scc4 lysate that flowed through the resin (lane 7), the wash (lane 8), and the imidazole elution of the ^15^N-Scc4:His_6_-Scc1 complex (lane 9). (**C**) SDS-PAGE analysis of the on-column association using purified ^15^N-Scc4 (lane 1) and co-expressed Scc4:His_6_-Scc1 (lane 2). Unlabeled Scc4 was eluted from the immobilized Scc4:His_6_-Scc1 complex (lane 3). The Ni-IMAC resin was washed, and the purified ^15^N-Scc4 sample was loaded onto the resin (the sample that flowed through the resin is shown in lane 4). Lane 5 is the sample that eluted from the Ni-IMAC resin using imidazole buffer. Scc4 (14.7 kDa) and His_6_-Scc1 (18.8 kDa) bands are indicated with an open and closed arrow head, respectively. Lane 10 in (**B**) and lane 6 in (**C**) are the Fisher BioReagents EZ-Run Rec pre-stained protein ladder with molecular weights listed to the right in kDa.

During our work to produce tag-free Scc4 by dissociating the co-expressed Scc4:His_6_-Scc1 complex on-column, we observed that the immobilized His_6_-Scc1 protein could be reused to capture Scc4 from lysate [22]. The on-column-associated complex was then eluted with imidazole buffer. This strategy (shown in Figure 3A) was used to produce the chain-selectively labeled complex, ^15^N-Scc4:His_6_-Scc1. Figure 3B shows the SDS-PAGE analysis of the steps in the process of associating ^15^N-Scc4 with unlabeled His_6_-Scc1 on the Ni-IMAC column. Sarkosyl was used to dissociate unlabeled Scc4 from the immobilized Scc4:His_6_-Scc1 complex (Figure 3B, lane 5) leaving His_6_-Scc1 immobilized on the Ni-IMAC resin (Figure 3B, lane 6). ^15^N-Scc4 in clarified lysate associated with the immobilized His_6_-Scc1, and the complex was eluted with imidazole (Figure 3B, lane 9). Interestingly, when the source of ^15^N-Scc4 was in buffer, the chain-selectively labeled complex did not associate on-column (the ^15^N-Scc4 flowed through the resin (Figure 3C, lane 4) and the imidazole elution only contained His_6_-Scc1 (Figure 3C, lane 5)). The on-column association was performed with purified Scc4 in both phosphate and Tris buffers with the same negative result [24], suggesting a role for lysate in the association. This role could be fulfilled by a specific lysate component or a general matrix effect on the structures of Scc4 and/or His_6_-Scc1 that facilitates binding [25].

**Figure 4:**
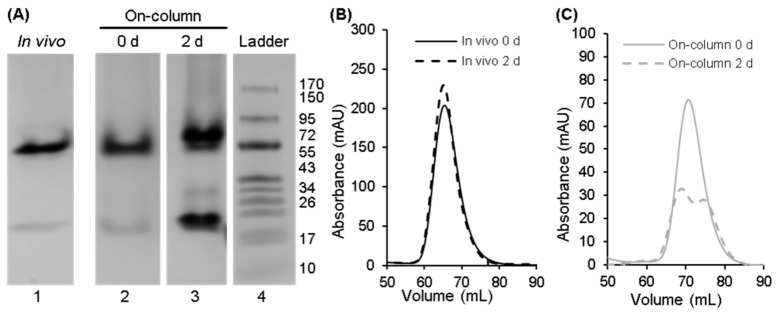
Native PAGE and qualitative SEC analyses to compare the stability of the in vivo-associated and on-column-associated complexes of Scc4:His_6_-Scc1. (**A**) Native PAGE gel of the in vivo-associated complex (lane 1) and the on-column-associated complex after purification (lane 2) and 2 days at room temperature (lane 3). (Native PAGE analysis of the in vivo-associated complex after 2 days at room temperature is shown in Figure 6, lane 2 from a second stability study.) Lane 4 is the Fisher BioReagents EZ-Run Rec pre-stained protein ladder with molecular weights listed to the right in kDa; the ladder was included as a qualitative guide. The full gels from which these lanes were extracted are shown in Figure S4. (**B**,**C**) Qualitative SEC analysis to assess the stability of (**B**) the in vivo-associated complex after purification (0 d, black solid line) and after 2 days at room temperature (dashed black line) and (**C**) the on-column-associated complex after purification (0 d, gray solid line) and after 2 days at room temperature (dashed gray line). Differences in the elution times between the (**B**) in vivo- and (**C**) on-column-associated complexes are not interpretable (refer to Section 2.5 for details).

The chain-selectively labeled complex made using ^15^N-Scc4 in lysate was analyzed using NMR and compared to the in vivo-associated complex to see if the native structure was reproduced using the on-column association method [24]. The ^1^H,^15^N-HSQC spectrum of the selectively labeled complex (published in Songok [24]) looked promising with peaks overlapping those of the in vivo complex, but it also showed peaks that matched ^15^N-Scc4. To determine if this result was due to contamination of the sample with excess ^15^N-Scc4 or dissociation of the complex over time, native gel analysis and qualitative SEC (Figure 4) were performed on the complex after its initial purification and 2 days at room temperature. The increase in the band intensity at the longer migration distance in the native gel (Figure 4A, lanes 2 and 3) and the appearance of a second peak in the chromatogram confirmed that the complex was dissociating over time (Figure 4C). In contrast, the in vivo-associated complex was stable with no change in its chromatograms (Figure 4B). It is not clear why the on-column-associated complex was less stable (especially since the initial NMR spectrum looked similar to the in vivo-associated spectrum). It is possible that subtle differences in the structure or dynamics destabilized the complex. Regardless, the on-column association of immobilized His_6_-Scc1 and Scc4 in lysate did not replicate the in vivo conditions to produce the native complex.

### 3.3. Purified Scc1-FLAG Interacts Weakly with Purified ^15^N-Scc4 Inducing Global Changes

**Figure 5:**
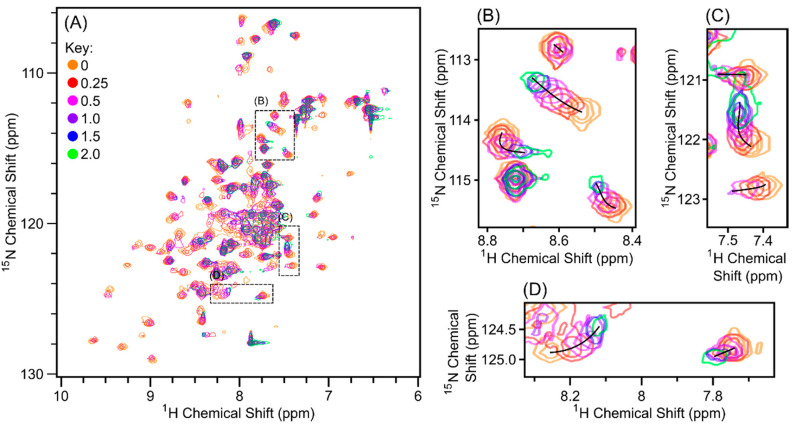
Overlay of ^1^H, ^15^N-HSQC spectra of ^15^N-Scc4 titrated with Scc1-FLAG. Relative moles of Scc1-FLAG to ^15^N-Scc4 added are 0 (orange), 0.25 (red), 0.5 (pink), 1.0 (purple), 1.5 (blue), and 2.0 (green). (**A**) The full spectra overlaid with boxes showing the expanded regions. (**B**–**D**) The expanded regions of the overlaid spectra in (**A**) with black lines marking the chemical shift perturbation over the course of the titration. The spectra were collected on a Bruker AVIII 500 MHz spectrometer with 8 scans for each spectrum, with the exception of the spectrum at 2.0 relative moles (green), which was collected with 32 scans. The contour levels were corrected for the number of scans to allow for direct comparison. Individual spectra are shown in Figure S5.

Since the on-column association method did not produce stable complexes, a simpler approach was tested: expressing and purifying each protein separately and recombining them in vitro. For the Scc4 and Scc1 proteins, Scc4 expresses well and is soluble, but Scc1 is insoluble when expressed alone [16]. The only soluble form of Scc1 that our group has successfully produced using *E. coli* is Scc1-FLAG after dissociation of the His_6_-Scc4:Scc1-FLAG complex immobilized on Ni-IMAC resin [22]. To determine if soluble Scc1-FLAG (prepared by on-column dissociation) and Scc4 associate in vitro to form the chain-selectively labeled ^15^N-Scc4:Scc1-FLAG complex, ^15^N-labeled Scc4 was titrated with unlabeled Scc1-FLAG and monitored with NMR. To confirm that adequate time was given to form the complex, the sample of ^15^N-Scc4 mixed with 2X molar Scc1-FLAG was analyzed again after 24 h and the spectrum did not change. Figure 5A shows an overlay of the ^1^H, ^15^N-HSQC spectra collected over the course of the titration (the individual spectra are shown in Figure S5). Nearly all of the peaks show chemical shift perturbations that are small (combined chemical shift changes < 0.4 ppm). Enlarged regions of the overlaid spectra are shown in Figure 5B–D to show the various peak perturbations observed. The characteristics of the chemical shift perturbations are not consistent with the association of a strong binding complex in slow exchange, which would show the ^15^N-Scc4 peaks decreasing and the ^15^N-Scc4:Scc1-FLAG peaks increasing with no chemical shift perturbations over the course of the titration. Instead, the perturbations indicate weak binding and are non-linear with peak broadening and a loss of peak dispersion. The non-linear shift means that binding is not a single binding mode, but something more complicated, such as multiple binding modes or a conformational change that alters the affinity between the two proteins [23]. The broadened and shifted peaks were identified using the Scc4 assignments [18] and mapped onto the Scc4 homology model (Figure S6). The changes do not map to a single area, indicating that the conformational changes induced by Scc1-FLAG are global. For single binding mode interactions, the global change would indicate an allosteric interaction, but the nature of the Scc1-FLAG and Scc4 association is more complicated and the observed effects can originate from direct binding, allosteric effects, induced aggregation, and/or induced unfolding [23]. We have not ruled out the possibility that the FLAG-tag contributes to the complexity of the titration results, especially since the FLAG-tag may affect the quality of the ^1^H, ^15^N-HSQC spectrum of the His_6_-Scc4:Scc1-FLAG complex (Figure 2B); however, the FLAG-tag does not interfere with the high-affinity association of the proteins in vivo, so it is unlikely to be the sole factor in inhibiting the association in vitro. Even though mechanistic details could not be gleaned from the titration data, it is clear that the in vitro interaction of purified Scc1-FLAG and Scc4 is weak and does not form the high-affinity in vivo-like complex.

### 3.4. Renatured, Chain-Selectively Labeled Scc4:His_6_-Scc1 Complexes Are Stable and Have In-Vivo-Like Structure

**Figure A1:**
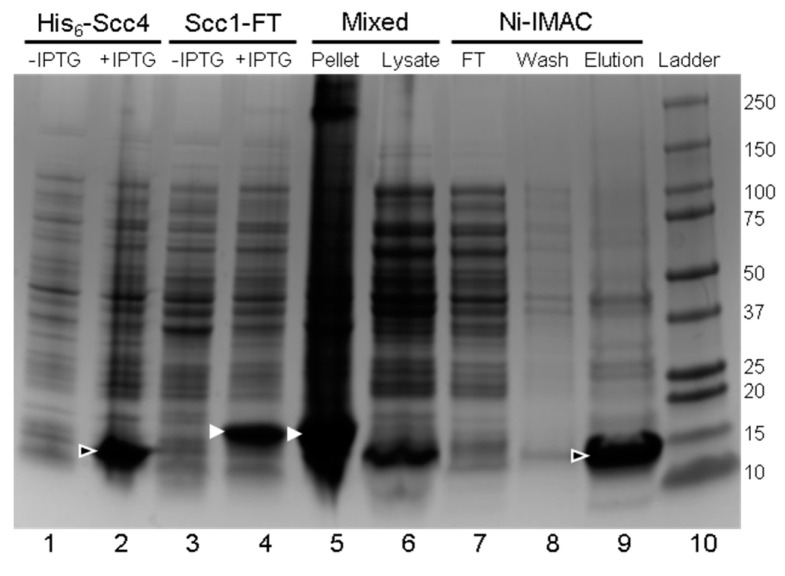
SDS-PAGE analysis of cell-cell mixture. Lanes are numbered at the bottom. Lanes 1 and 2 and lanes 3 and 4 are proteins in the cultures before (-IPTG) and after (+IPTG) induction of His_6_-Scc4 and Scc1-FLAG, respectively. Lanes 5 and 6 are the pellet and clarified lysate produced after the cells were mixed and lysed together. Lanes 7, 8, and 9 are the lysate that flowed through the Ni-IMAC resin (FT), buffer wash step, and imidazole elution, respectively. The arrow heads indicate bands for Scc1-FLAG (18.2 kDa) and His_6_-Scc4 (15.8 kDa). Lane 10 is the protein molecular weight marker (Bio-Rad Precision Plus protein ladder) with molecular weights listed to the right in kDa.

**Figure A2:**
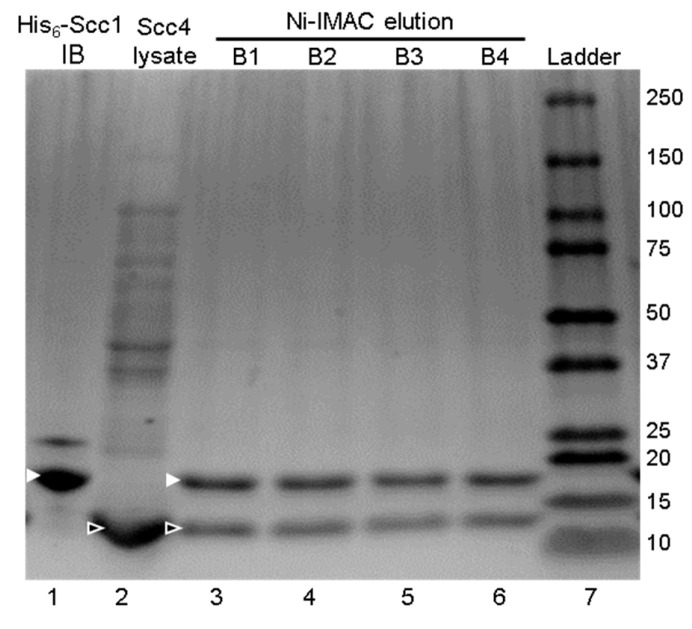
SDS-PAGE analysis of the association of denatured His_6_-Scc1 and Scc4 in lysate using different buffer conditions. Lane 1 is the purified His_6_-Scc1 inclusion body (IB), and lane 2 is clarified cell lysate containing Scc4. Denatured His_6_-Scc1 was diluted with 4 different buffers (B1–B4, described in Section 2.6) before mixing dropwise into the Scc4 lysate. Each mixture was purified using Ni-IMAC to determine if the complex formed; lanes 3–6 are the Ni-IMAC elution samples. The arrow heads indicate bands for His_6_-Scc1 (18.8 kDa) and Scc4 (14.7 kDa). Lane 7 is the Bio-Rad Precision Plus protein ladder with molecular weights listed in kDa to the right.

Based on the results that the proteins did not associate properly when Scc1 was soluble (in buffer or on-column) or insoluble in lysate (Appendix A, Figure A1), we sought to mix the two proteins with Scc1 in a denatured state. Purified His_6_-Scc1 inclusion bodies (Figure S7) were denatured and added dropwise to clarified lysate containing Scc4 in a ratio of 1 part culture volume His_6_-Scc1 to 3.5 parts culture volume Scc4 (Appendix B). The renatured protein complex was purified using Ni-IMAC. SDS-PAGE analysis of the Ni-IMAC elution samples (Figure A2, lanes 3–6) shows the successful association of the Scc4:His_6_-Scc1 complex with a 1:1 molar ratio.

**Figure 6:**
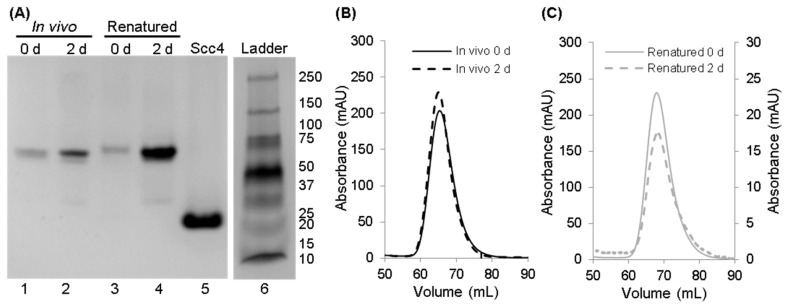
Native PAGE and qualitative SEC analyses to compare the stability of the in vivo-associated and renatured complexes of Scc4:His_6_-Scc1. (**A**) Native PAGE of the in vivo-associated complex (lanes 1 and 2) and the renatured complex (lanes 3 and 4) after purification and after 2 days at room temperature. Lane 5 is Scc4 for comparison. Lane 6 is the Bio-Rad Precision Plus protein ladder with molecular weights listed to the right in kDa; the ladder was included as a qualitative guide. The full gel from which these lanes were extracted is shown in Figure S8. (**B**,**C**) Qualitative SEC analysis to assess the stability of (**B**) the in vivo-associated complex after purification (0 d, black solid line) and after 2 days at room temperature (dashed black line) and (**C**) the renatured complex after purification (0 d, gray solid line) and after 2 days at room temperature (dashed gray line). (**C**) The right vertical axis corresponds to the renatured complex after 2 days; a smaller amount was analyzed to reverse the bulk of the sample for NMR analysis. Differences in the elution times between the (**B**) in vivo- and (**C**) on-column-associated complexes are not interpretable (refer to Section 2.5 for details).

The renatured Scc4:His_6_-Scc1 complex was tested for stability by leaving the purified sample at room temperature for 2 days and analyzing the sample by native PAGE and qualitative SEC. In vivo-associated Scc4:His_6_-Scc1 was treated the same for comparison. Figure 6A shows the results of the native PAGE analysis of the complexes on days 0 and 2 (lanes 1–4). Purified Scc4 (Figure 6A, lane 5) was included as a migration reference. Both complexes showed prominent bands at shorter migration distances compared to Scc4, indicating that the proteins were migrating as a complex. After 2 days at room temperature, there was no change in the band pattern for the in vivo or renatured complexes. The qualitative SEC analyses of the in vivo complex (Figure 6B) and the renatured complex (Figure 6C) also show that the complexes are stable with no change in the retention volumes from day 0 to day 2.

Chain-selectively labeled ^15^N-Scc4:His_6_-Scc1 and Scc4:^15^N-His_6_-Scc1 were prepared using the renaturing method for NMR analysis. In these experiments, 1 part culture volume of His_6_-Scc1 was mixed with 2 parts culture volume of ^15^N-Scc4 to provide excess His_6_-Scc1. The excess His_6_-Scc1 ensured that all of the ^15^N-Scc4 was bound (excess His_6_-Scc1 precipitated and was removed by centrifugation from the final mixture before Ni-IMAC purification). The SDS-PAGE analysis confirmed the association of the ^15^N-Scc4:His_6_-Scc1 complex (Figure S9A, lane 7) in the Ni-IMAC elution. The yield was 6–8 mg per L (of ^15^N-Scc4) culture, which was comparable to the 10–12 mg per L culture of co-expressed, in vivo-associated Scc4:His_6_-Scc1 complex. The Scc4:^15^N-His_6_-Scc1 complex was made using the same method with similar results (Figure S9B, lane 7) and a yield of 6–8 mg per L culture. The imidazole elution samples for both complexes were further purified by SEC in the same manner as the in vivo-associated complex (Figure S9A, lanes 9 and Figure S9B, lanes 9).

**Figure 7:**
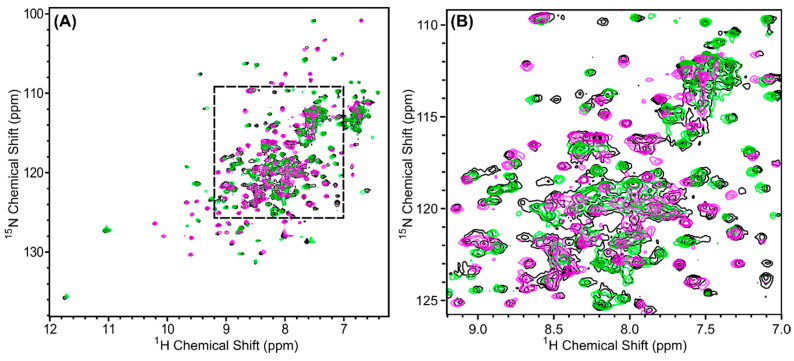
^1^H, ^15^N-HSQC spectra of in vivo-associated ^15^N-labeled Scc4:His_6_-Scc1 (black), renatured ^15^N-Scc4:His_6_-Scc1 (pink), and renatured Scc4:^15^N-His_6_-Scc1 (green) overlaid to compare the peak positions and intensities. (**A**) The full spectra with a dashed box indicating the region expanded in (**B**). Over 97% of the peaks from the renatured complexes and the in vivo-associated complex overlap. The minor difference observed in (**B**) are discussed in Appendix C, Figure A3.

^1^H,^15^N HSQC spectra of the chain-selectively labeled complexes were collected and compared to the spectrum of the in vivo-associated (fully ^15^N-labeled) complex. To investigate any differences between the renatured complexes and the in vivo-associated complex, the HSQC spectra were overlaid to compare the peak positions and intensities. Figure 7A shows that over 97% of the peaks overlap verifying that the two quaternary structures are the same and validating the suitability of the renaturing method to pursue the high-resolution 3-dimensional structure of the complex by NMR.

**Figure 8:**
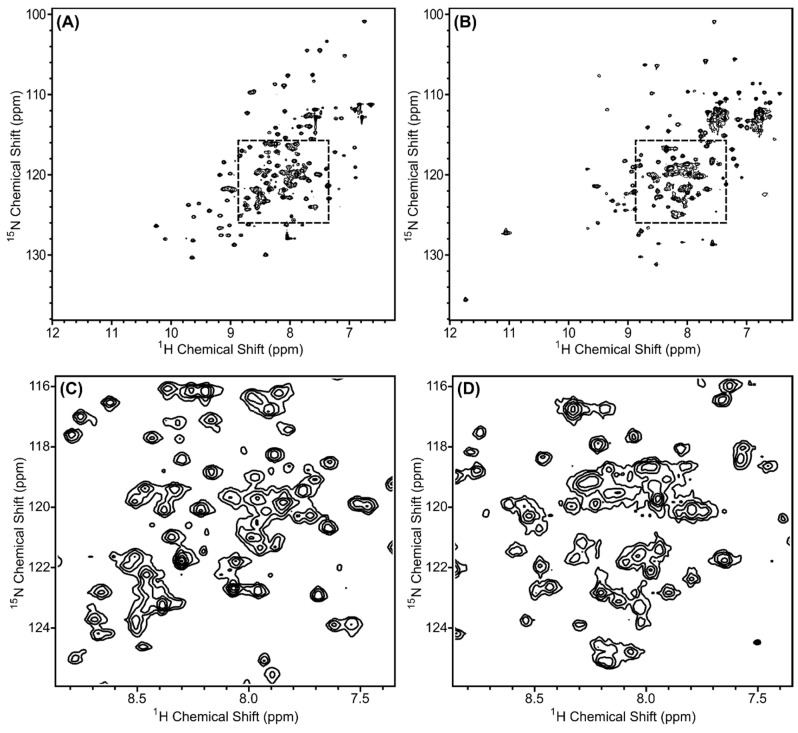
^1^H, ^15^N-HSQC spectra of renatured (**A**,**C**) ^15^N-Scc4:His_6_-Scc1 and (**B**,**D**) Scc4:^15^N-His_6_-Scc1 complexes. The regions shown in (**C**,**D**) are expanded from the dashed boxes shown in (**A**,**B**), respectively. All samples were 0.35 mM concentration and collected on a Bruker 500 MHz spectrometer with 64 scans.

**Figure 9:**
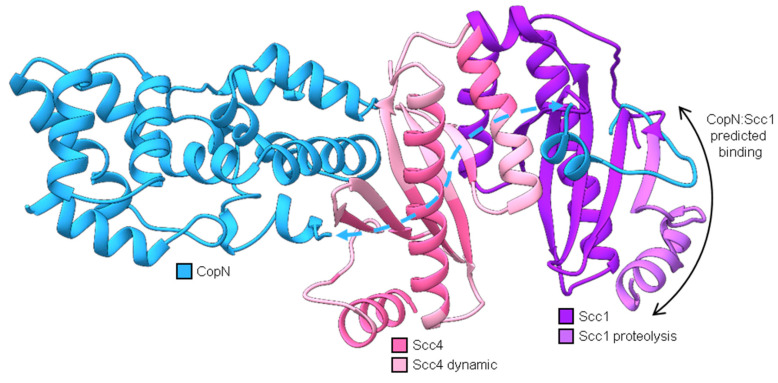
Structural homology models (generated using the protein homology/analogy recognition engine, Phyre2 [26]) of CopN (blue), Scc4 (pink), and Scc1 (purple) aligned on the X-ray structure of *Yersinia pestis* YopN:SycN:YscB complex (Protein Data Bank code 1XKP) [16,27]. Phyre2 alignments are shown in Figures S10, S11, and S12. Models for CopN include two fragments (residues 37–60 and 77–267), which are connected with a dashed blue arrow. The *N*-terminal fragment of CopN was predicted to interact with the first beta strand of Scc1 (black double arrow). As part of the Scc4:Scc1 complex, this region of Scc1 (residues 1–31, light purple) was susceptible to proteolytic cleavage [16]. The regions of Scc4 in light pink are residues determined to be dynamic and form random coils in Scc4 [18].

By using the chain-selective labeling approach, the spectra of the complex were simplified and each protein chain was analyzed separately. Figure 8A,B show the ^1^H, ^15^N-HSQC spectra of ^15^N-Scc4:His_6_-Scc1 and Scc4:^15^N-His_6_-Scc1, respectively. As expected, the number of peaks in the spectra are reduced compared to the fully ^15^N-labeled complex (Figure 2A) with only Scc4 peaks visible in Figure 8A,C (143 peaks) and His_6_-Scc1 peaks in Figure 8B,D (165 peaks). Since His_6_-Scc1 is insoluble alone, Figure 8B is the first recorded ^1^H, ^15^N-HSQC spectrum of His_6_-Scc1. The number of peaks (backbone and sidechain amides) in each chain-selectively labeled spectrum represents 97% and 76% of the expected peaks for Scc4 and His_6_-Scc1, respectively, with a total of 308 peaks. The total number of peaks is consistent with the 298 peaks counted for the in vivo-associated complex with 10 additional peaks identified due to the reduced resonance overlap. NMR analysis of the selectively labeled complexes shows that Scc4 is well-structured in the complex with a high percentage of expected peaks (Figure 8A), but 24% of the expected peaks for His_6_-Scc1 (approximately 40 residues) have low intensity or are missing (Figure 8B), indicating that a portion of His_6_-Scc1 is dynamic. Based on our previous work, the *N*-terminus of His_6_-Scc1 (in the co-expressed Scc4:His_6_-Scc1 complex), but not the *C*-terminus, was susceptible to limited proteolysis (Figure 9, light purple) [16]. The His_6_-tag and *N*-terminal Scc1 residues are likely the dynamic region with missing peaks in the NMR spectrum. This Scc1 *N*-terminal region was also predicted to bind CopN (Figure 9) based on structural homology [16]. The dynamic nature of the Scc1 *N*-terminus suggests that it binds CopN in an induced fit model, regulating CopN recognition and release (secretion) by the T3SS chaperone complex. The chain-selective labeling approach will allow future work to verify the identity of the missing His_6_-Scc1 resonances, study CopN recognition using NMR, and determine the high-resolution structures of Scc4 and His_6_-Scc1 in the complex.

### 3.5. Different Structures for Scc4′s Different Functions

**Figure 10:**
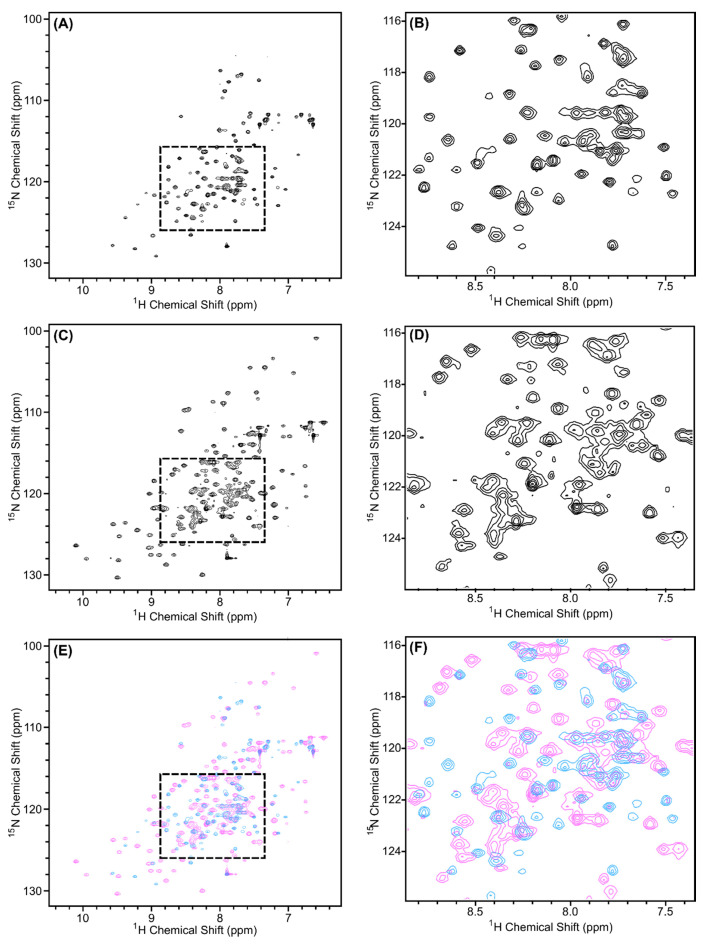
^1^H, ^15^N-HSQC spectra of (**A**,**B**) ^15^N-Scc4 and (**C**,**D**) Scc4 in the ^15^N-Scc4:His_6_-Scc1 renatured complex. (**E**,**F**) Overlay of the ^1^H,^15^N-HSQC spectra of ^15^N-Scc4 (blue) and Scc4 in the ^15^N-Scc4:His_6_-Scc1 renatured complex (pink). (**B**,**D**,**F**) Expanded regions from dashed boxes in (**A**,**C**,**E**), respectively.

One of the questions our group is pursuing is the mechanism by which Scc4 switches between its functions as a T3SS chaperone and an RNAP-binding protein. During the developmental cycle, Scc4 temporally binds Scc1/CopN and σ^66^RNAP, and the structure of Scc4 must accommodate association into these two different complexes. Comparing the backbone structure of Scc4 with the Scc4:Scc1 T3SS chaperone complex provides insight into the switching mechanism. From our previous work with Scc4, the NMR resonance assignments, secondary structure, and disorder parameters for Scc4 revealed a dynamic protein (56% random coil) with unassigned residues across the sequence and disordered regions where Scc1 is predicted to bind Scc4 (Figure 9, light pink residues) [18]. In contrast, the homology model of Scc4 constructed from the X-ray structures of other T3SS chaperone complexes in Figure 9 predicted a compact tertiary structure with few random coil regions (35% random coil) [16]. In Figure 10, the ^1^H, ^15^N-HSQC spectra of Scc4 and ^15^N-Scc4:His_6_-Scc1 are shown and overlaid for comparison. Comparing Figure 10A,C, Scc4 in the complex, ^15^N-Scc4:His_6_-Scc1, has a larger peak dispersion, more peaks, and more uniform peak intensity compared to Scc4 alone. All three of these characteristics indicate that Scc4 in the complex has more residues participating in structural elements with fewer regions of local mobility on the ms to μs timescale (broad peaks) and fewer dynamic, unstructured regions with sub-ns timescale motions (sharp, high intensity peaks) [28]. With 97% of the expected peak count in the ^15^N-Scc4:His_6_-Scc1 spectrum, the disordered regions of Scc4 clearly became well-structured in the T3SS chaperone complex. The overlaid spectra (Figure 10E,F) show very little (<15%) peak overlap, indicating that at least 85% of the backbone structures of Scc4 (free and bound) are completely different. This result is surprising considering only 28% of Scc4′s residues are predicted to be within 5Å of the Scc1 interface (Figure S13). Even if the assumption is made that the overlapping ^15^N-Scc4:His_6_-Scc1 peaks have the same resonance assignments as Scc4 alone, the residues assigned to these peaks do not cluster to a specific region of the protein when mapped onto the homology model (Figure S13) as would be expected if part of the structure was unchanged. Given the extent and fragmented nature of the peak shifts, it can be concluded that the entire tertiary structure of Scc4 changed upon binding Scc1. These results are consistent with the NMR-derived dynamic regions of Scc4 (Figure 9, light pink residues) becoming structured upon binding Scc1 as predicted by the T3SS chaperone homology model (Figure 9, all pink residues). This elasticity allows Scc4 to change its structure and bind different complexes depending on the availability of its binding partners. It also explains why CopN does not bind Scc4, but only Scc4 in complex with Scc1. Unlike the disordered region of Scc1 that was predicted to bind CopN, the Scc4 predicted-interface with CopN is well-structured like a lock-and-key binding model.

## 4. Conclusions

The results from this work provide an approach for producing chain-selectively labeled Scc4:His_6_-Scc1 complex for NMR analysis and new models for Scc4 and Scc1 association, Scc4′s switching mechanism, and CopN recognition. The labeling approach was sucessful in producing the complex with the correct structure and in yields (6–8 mg per L culture) sufficient for NMR analysis. The reduced resonance overlap allows the pursuit of the Scc4:Scc1 T3SS complex structure by NMR. Analysis of the chain-selectively labeled complexes provided new information on the nature of each protein in the complex. Scc1 has a dynamic region hypothesized to be the *N*-terminal region and bind CopN. Conversely, Scc4 was well-structured in the complex compared to its highly dynamic free form, and the backbone structure of Scc4 completely changed upon binding Scc1. The molecular mechanisms of this structural switch can now be studied using the chain selective labeling approach.

**Figure 11:**
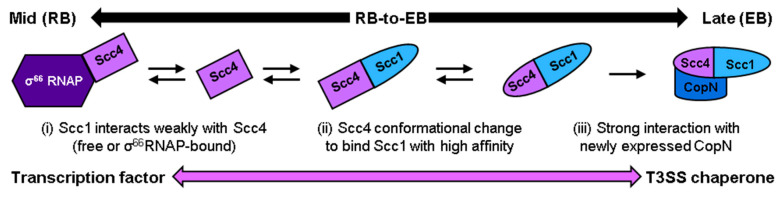
Working model of Scc4′s functional switch from a transcription factor to a T3SS chaperone during the mid-to-late stages of development. Newly expressed Scc1 is proposed to (**i**) interact with Scc4 in its free form or bound to σ^66^RNAP and (**ii**) induce a change in Scc4′s structure that leads to the association of Scc4 and Scc1, which may require other factors based on our results. (**iii**) Once the high affinity Scc4:Scc1 T3SS complex is formed, it binds newly expressed CopN.

A new working model for Scc4′s switch from a transcription factor to a T3SS chaperone, where conformational changes of both Scc4 and Scc1 are required to produce the native complex, was informed by the results from different methods of associating Scc4 and Scc1. The association of Scc4 and Scc1 under non-denaturing conditions, such as the on-column association of Scc4 and His_6_-Scc1, required *E. coli* lysate. Many *E. coli* lysate components are the same or homologous to *CT*’s, so it is possible that a molecular chaperone, co-factor, or modulator was involved in the proteins’ association and folding. The titration of Scc4 with Scc1-FLAG induced changes in Scc4, but the high-affinity association was mitigated until Scc1 was at least partially unfolded in 2 M GuHCl. Taken together, a new working model is proposed (Figure 11), where the interaction of Scc4 by Scc1 plays a role in Scc4′s functional switch from a transcription factor to a T3SS chaperone during the mid-to-late stages of the developmental cycle. We hypothesize that newly expressed Scc1 in *CT* interacts with Scc4 either in its free form or when it is bound to σ^66^RNAP (Figure 11, i), inducing a change in Scc4′s structure (Figure 11, ii). The association of Scc4 and Scc1 to form the T3SS chaperone complex (Figure 11, ii) may involve other components to partially unfold Scc1, such as a molecular chaperone. Once the complex of Scc4 and Scc1 is formed, it binds newly expressed CopN with high affinity (Figure 11, iii), poising the essential virulence effector for secretion upon contact with the next host cells.

## Figures and Tables

**Figure A3:**
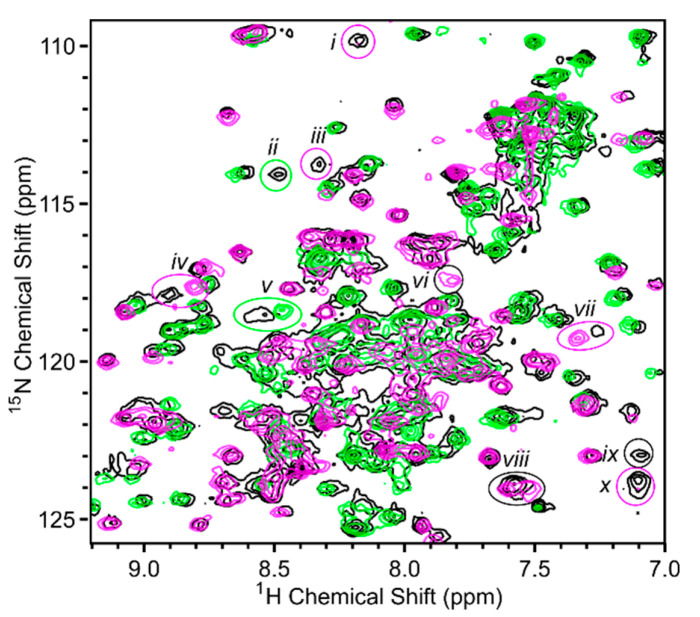
The expanded region of ^1^H,^15^N-HSQC spectra of in vivo-associated ^15^N-labeled Scc4:His_6_-Scc1 (black), renatured ^15^N-Scc4:His_6_-Scc1 (pink), and renatured Scc4:^15^N-His_6_-Scc1 (green) overlaid to highlight minor differences in the spectra. The region shown is the same as in Figure 7B. Minor peak differences between the spectra are indicated with circles labeled i-x. Pink and green circles indicate the assignment of the peak to the Scc4 or His_6_-Scc1 chain, respectively. Black circles show peaks unique to either the in vivo-associated or renatured complex spectrum.

## Supplementary Materials

The following are available online at https://www.mdpi.com/2218-273X/10/11/1480/s1, Figure S1: Expression and purification of ^15^N-labeled Scc4:His_6_-Scc1, Figure S2: Expression and purification of ^15^N-labeled His_6_-Scc4:Scc1-FLAG, Figure S3: Overlay of the ^1^H-^15^N HSQC spectra of the in vivo-associated, ^15^N-labeled protein complexes, Figure S4: Full gel images of the native gels from Figure 4A, Figure S5: ^1^H,^15^N-HSQC spectra of ^15^N-Scc4 titrated with Scc1-FLAG, Figure S6: ^1^H,^15^N-HSQC peak changes during the Scc1-FLAG titration of Scc4 mapped on the Scc4 homology model, Figure S7: SDS-PAGE analysis of the His_6_-Scc1 inclusion body purification, Figure S8: Full gel images of the native gels from Figure 6A, Figure S9: SDS-PAGE analysis of the renatured and chain-selectively labeled Scc4:His_6_-Scc1 complexes, Figure S10: Phyre2 alignment of *CT* Scc4 and *Yersinia pestis* YscB from PDB 1XKP, chain C, Figure S11: Phyre2 alignment of *CT* Scc1 and *Yersinia pestis* SycN from PDB 1XKP, chain B, Figure S12: Phyre2 alignment of *CT* CopN and *Yersinia pestis* YopN from PDB 1XKP, chain A, Figure S13: Scc4 homology model with Scc1 interface and overlapping resonances.

## Appendix A

Since in vivo conditions may be required to form the Scc4:Scc1 complex, an alternative approach was tested to produce the chain-selectively labeled complex by combining cells containing expressed His_6_-Scc4 with cells containing expressed Scc1-FLAG immediately before lysis and purification. Even though Scc1-FLAG is insoluble when expressed alone, the strategy combines the two proteins in the presence of cell lysate. Purification of the mixed cell lysate by Ni-IMAC should afford His_6_-Scc4 and Scc1-FLAG if the proteins associate strongly during lysis. Figure A1, lane 9 shows the SDS-PAGE analysis of the elution containing only His_6_-Scc4 and no Scc1-FLAG. Inspection of the lysis pellet (lane 5) and clarified lysate (lane 6) indicates that Scc1-FLAG remained insoluble even in the presence of soluble His_6_-Scc4. Similar to the results of mixing the pure proteins, mixing the proteins during lysis did not afford the tightly associated complex. This result demonstrates that insoluble Scc1-FLAG is intractable to association with Scc4 even in the presence of lysate.

## Appendix B

Inclusion bodies of His_6_-Scc1 from a 500 mL expression culture of *E. coli* were purified (Figure S7) and denatured in 6 M GuHCl Tris buffer. The concentration of GuHCl was diluted with buffer to a final concentration of 2 M, which was determined to be the minimum concentration necessary to maintain His_6_-Scc1 solubility (based on visual turbidity). The denatured, soluble His_6_-Scc1 was added dropwise to clarified lysate containing Scc4. Ideally, equal moles of His_6_-Scc1 and Scc4 would be mixed, but the concentrations of the proteins could not be determined due to the complicated sample matrix. The expression of His_6_-Scc1 alone typically has a higher yield than Scc4, so we related the concentrations of the proteins to the culture volumes, which was 1 part His_6_-Scc1 to 3.5 parts Scc4. After His_6_-Scc1 was added to Scc4, the final concentration of GuHCl was 0.75 M and the mixture was purified using Ni-IMAC. Figure A2 shows the SDS-PAGE results of testing four buffers (refer to Section 2.6 for details) to dilute the denatured His_6_-Scc1 protein solutions before mixing with the Scc4 lysate. The Ni-IMAC elution samples showed successful association of the Scc4:His_6_-Scc1 complex with a 1:1 molar ratio and similar yields for all buffers (Figure A2, lanes 3–6). The additional components of GSH/GSSG and l-arginine to the buffer did not impact the association.

## Appendix C

Over 97% of the peaks in the HSQC spectra of the renatured complexes overlap with peaks of the in vivo-associated complex (Figure 7A,B). The few exceptions are highlighted in Figure A3. Three peaks in the spectra have small chemical shift differences such that the peaks do not overlap (see iv, v, vii in Figure A3). Other peaks have lower relative intensity in the renatured spectra, such as i, ii, iii, and x in Figure A3. The presence of these peaks were verified by lowering the contour levels of the respective chain-selectively labeled spectrum (i, iii, x belong to Scc4 and ii belongs to His_6_-Scc1). Finally, there are two peaks in the ^15^N-Scc4:His_6_-Scc1 spectrum (vi and viii) and one peak in the in vivo-associated complex spectrum (ix) that do not have an obvious counterpart. Overall, these data indicate minor differences in the overall structure and dynamics of the complexes. We plan to identify and characterize these differences in the future by assigning the backbone resonances of the renatured complexes.
