# Supplementary material for: Chain-Selective Isotopic Labeling of the Heterodimeric Type III Secretion Chaperone, Scc4:Scc1, Reveals the Total Structural Rearrangement of the Chlamydia trachomatis Bi-Functional Protein, Scc4
Source: Biomolecules. 2020 Oct 24;10(11):1480. doi: 10.3390/biom10111480 (PMC7692554; doi:10.3390/biom10111480)
Supplement: Supplementary file 1 [file biomolecules-10-01480-s001.pdf]

Chain-selective Isotopic Labeling of the Heterodimeric Type III Secretion Chaperone, Scc4:Scc1, Reveals the Total Structural Rearrangement of the *Chlamydia trachomatis* Bi-functional Protein, Scc4

Thilini O. Ukwaththage[1], Samantha M. Keane,[1] Li Shen[2] and Megan A. Macnaughtan[1,*]

[1] Department of Chemistry, Louisiana State University, Baton Rouge, LA 70803, USA
[2] Department of Microbiology, Immunology, and Parasitology, Louisiana State University Health Sciences Center, New Orleans, LA 70112, USA
* Correspondence: macnau@lsu.edu

Supplementary Materials

Figure S1: Expression and purification of ^{15}N-labeled Scc4:His$_6$-Scc1 2

Figure S2: Expression and purification of ^{15}N-labeled His$_6$-Scc4:Scc1-FLAG 2

Figure S3: Overlay of the 2D ^{1}H-^{15}N HSQC spectra of the *in vivo*-associated, ^{15}N-labeled protein complexes 3

Figure S4: Full gel images of the native gels from Figure 4A 3

Figure S5: ^{1}H,^{15}N-HSQC spectra of ^{15}N-Scc4 titrated with Scc1-FLAG 4

Figure S6: ^{1}H,^{15}N-HSQC peak changes during the Scc1-FLAG titration of Scc4 mapped on the Scc4 homology model 5

Figure S7: SDS-PAGE analysis of the His$_6$-Scc1 inclusion body purification 6

Figure S8: Full gel images of the native gels from Figure 6A 6

Figure S9: SDS-PAGE analysis of the renatured and chain-selectively labeled Scc4:His$_6$-Scc1 complexes 7

Figure S10: Phyre2 alignment of *CT* Scc4 and *Yersinia pestis* YscB from PDB 1XKP, chain C 7

Figure S11: Phyre2 alignment of *CT* Scc1 and *Yersinia pestis* SycN from PDB 1XKP, chain B 8

Figure S12: Phyre2 alignment of *CT* CopN and *Yersinia pestis* YopN from PDB 1XKP, chain A 9

Figure S13: Scc4 homology model with Scc1 interface and overlapping resonances 10

References 10

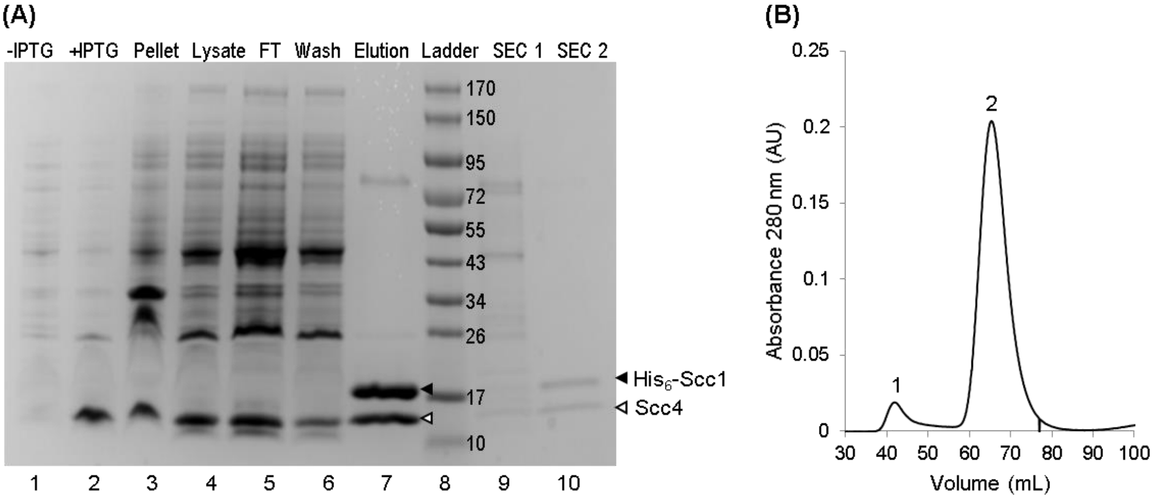

Figure S1. Expression and purification of ^{15}N-labeled Scc4:His_6-Scc1. (A) SDS-PAGE analysis with lanes numbered at the bottom. Lanes 1 and 2 are proteins in the culture before (-IPTG) and after (+IPTG) induction. Lanes 3 and 4 are the pellet and clarified lysate after cell lysis and centrifugation. Lanes 5, 6, and 7 are the lysate that flowed through the Ni-IMAC resin (FT), buffer wash step, and elution of the Scc4:His_6-Scc1 complex using imidazole buffer, respectively. Lane 8 is the Fisher BioReagents EZ-Run Rec pre-stained protein ladder with molecular weights listed to the right in kDa. Lanes 9 and 10 are samples from the SEC purification of the complex shown in (B). The arrow heads indicate the bands corresponding to Scc4 (14.7 kDa) and His_6-Scc1 (18.8 kDa). (B) SEC purification of the Scc4:His_6-Scc1 imidazole elution produced two peaks labeled 1 and 2. The SDS-PAGE analysis of these peaks are shown in lanes 9 and 10, respectively, in (A). The second peak contains the purified Scc4:His_6-Scc1 complex.

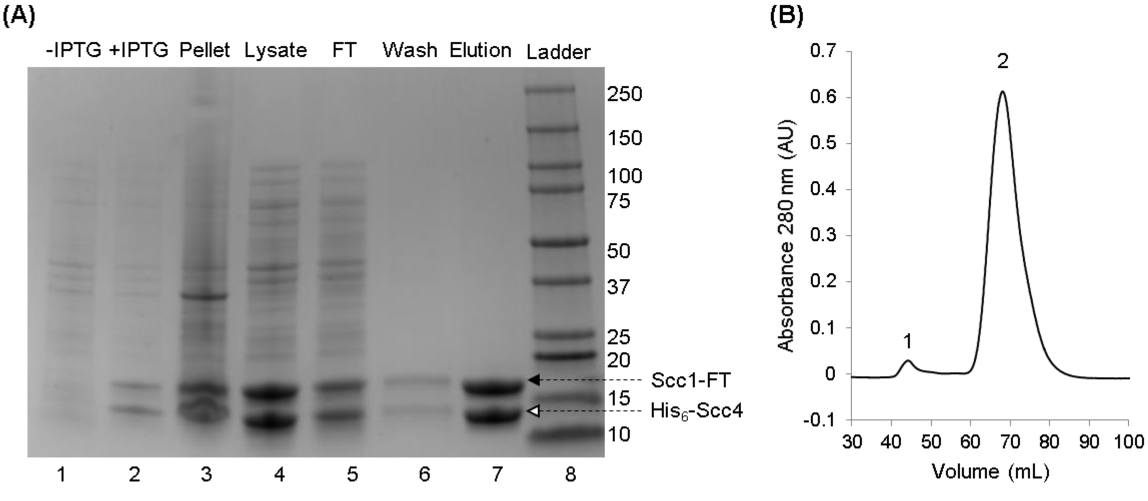

Figure S2. Expression and purification of ^{15}N-labeled His_6-Scc4:Scc1-FLAG. (A) SDS-PAGE analysis with lanes numbered at the bottom. Lanes 1 and 2 are proteins in the culture before (-IPTG) and after (+IPTG) induction. Lanes 3 and 4 are the pellet (Pellet) and clarified lysate (Lysate) after cell lysis and centrifugation. Lanes 5, 6, and 7 are the lysate that flowed through the Ni-IMAC resin (FT), buffer wash step (Wash), and elution of the His_6-Scc4:Scc1-FLAG complex using imidazole buffer (Elution), respectively. Lane 8 is the Bio-Rad Precision Plus protein ladder with molecular weights listed to the right in kDa. The arrow heads indicate the bands corresponding to His_6-Scc4 (15.8 kDa) and Scc1-FLAG (18.2 kDa). (B) SEC purification of the His_6-Scc4:Scc1-FLAG imidazole elution produced two peaks labeled 1 and 2. The second peak contains the purified His_6-Scc4:Scc1-FLAG complex.

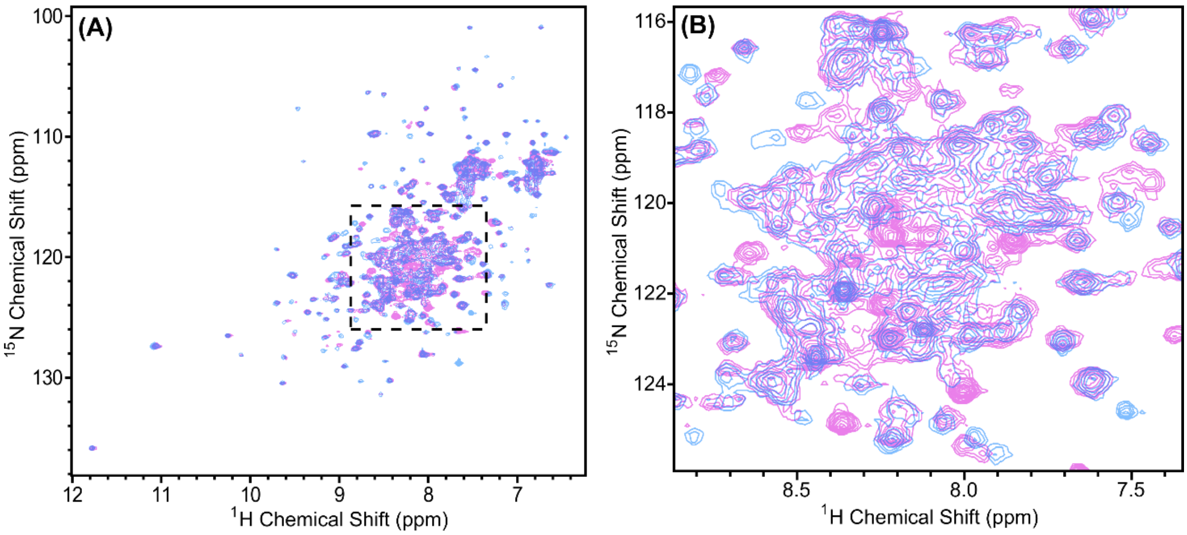

Figure S3. 2D ^{1}H-^{15}N HSQC spectra of the *in vivo*-associated, ^{15}N-labeled protein complexes Scc4:His$_6$-Scc1 (blue) and His$_6$-Scc4:Scc1-FLAG (pink) with the (A) full spectra and (B) expanded regions from the dashed box in (A). The complexes were 0.35 mM concentration in 50 mM sodium phosphate, 10 mM DTT, pH 7.3 buffer, and the spectra were collected with 32 scans using a Bruker AVIII 500 MHz spectrometer.

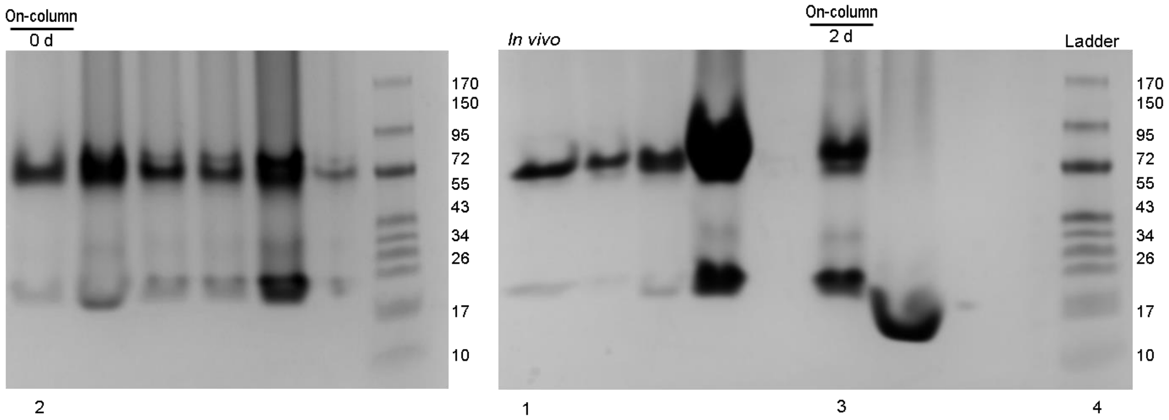

Figure S4. Full gel images of the native gels from Figure 4A. Lane numbers across the bottom correspond to the lane numbers in Figure 4A.

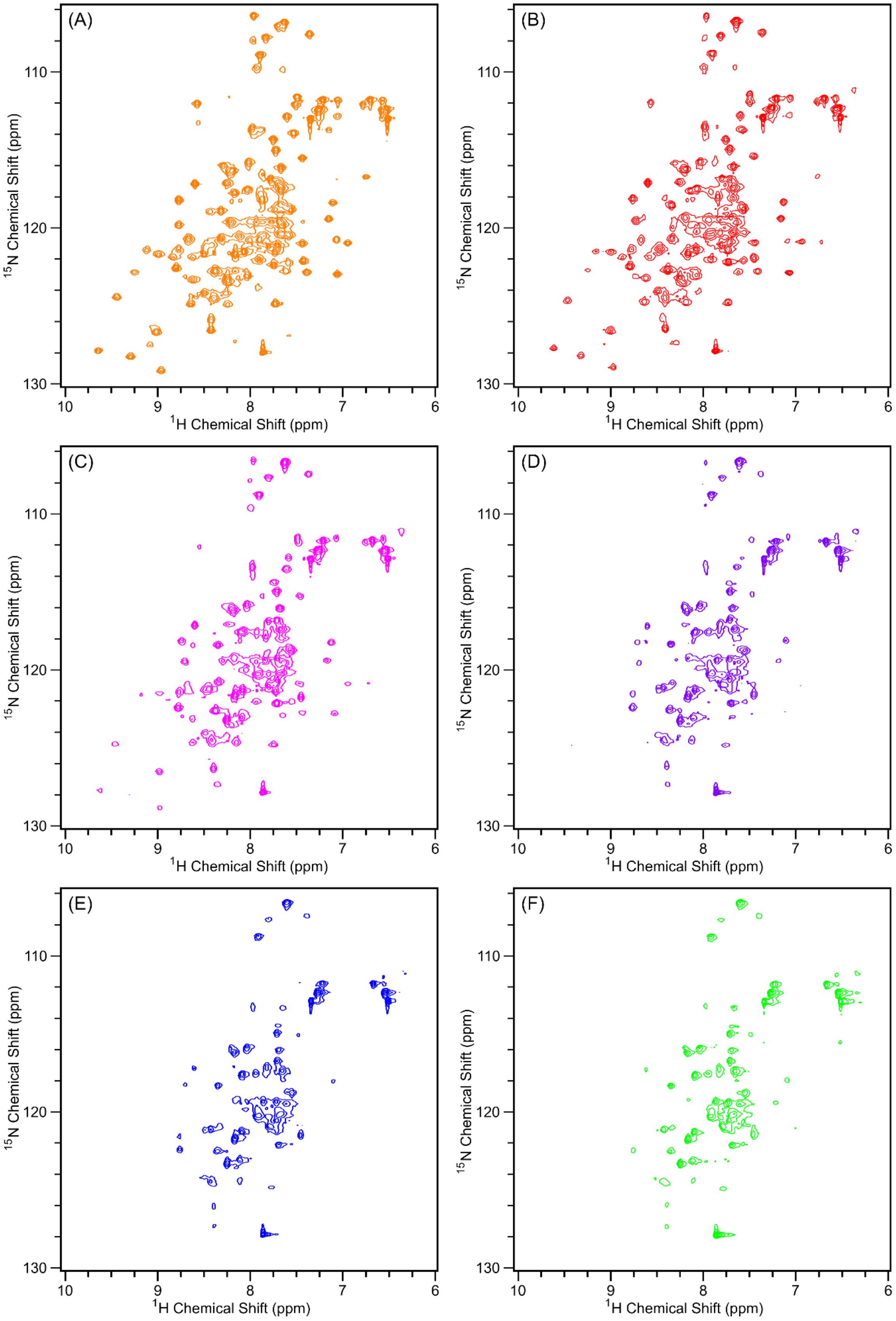

Figure S5. (A-F) ^{1}H,^{15}N-HSQC spectra of ^{15}N-Scc4 titrated with Scc1-FLAG. Relative moles of Scc1-FLAG to ^{15}N-Scc4 added are (A) 0, (B) 0.25, (C) 0.5, (D) 1.0, (E) 1.5, and (F) 2.0. The spectra were collected on a Bruker AVIII 500 MHz spectrometer with 8 scans for each spectrum, with the exception

of spectrum (F), which was collected with 32 scans. The contour levels were corrected for the number of scans to allow for direct comparison.

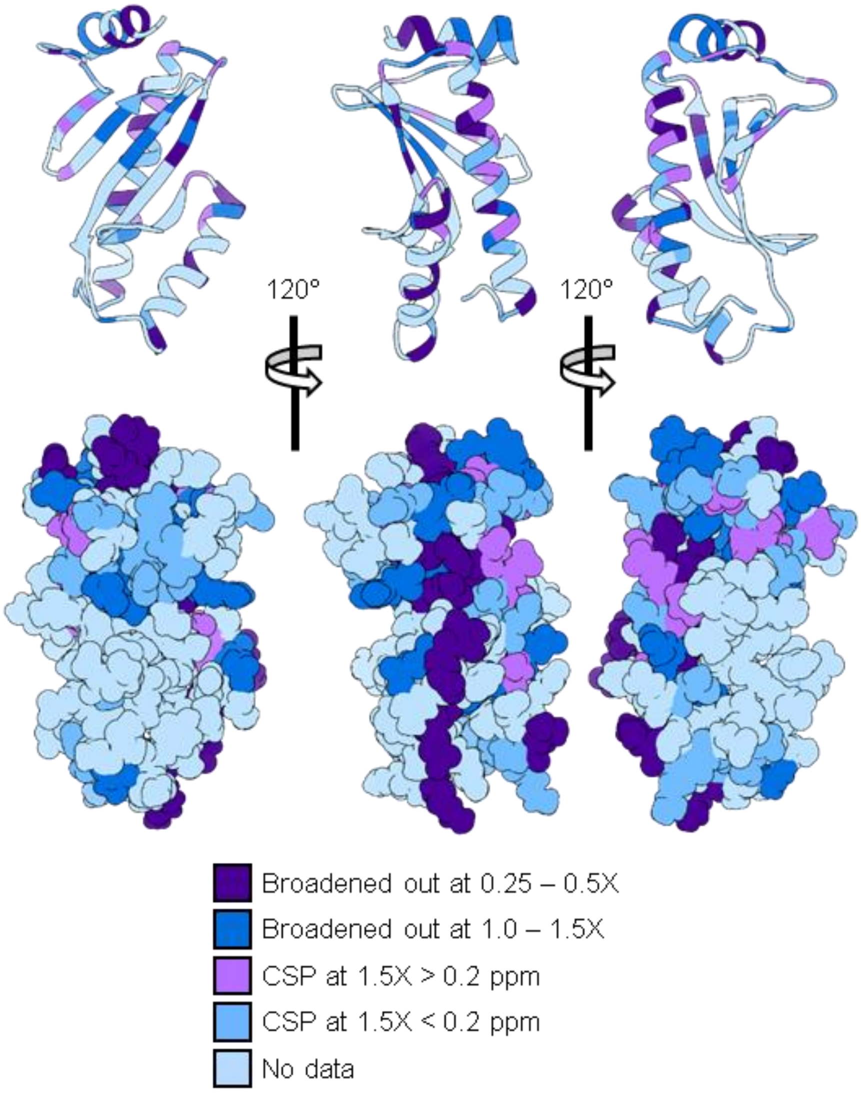

Figure S6. ^{1}H,^{15}N-HSQC peak changes during the Scc1-FLAG titration of Scc4 mapped onto the Scc4 homology model. The homology model is shown as a ribbon diagram (top) and in all-atom representation (bottom) with three points of view via rotation about the vertical axis shown. The HSQC peaks were identified using the published assignments [1]. Light blue residues indicate peaks that are unassigned [1] or cannot be positively identified or tracked due to overlap during the titration. The disappearance of peaks due to broadening at 0.25 – 0.5X addition of Scc1-FLAG are shaded dark purple and at 1.0 – 1.5X addition of Scc1-FLAG are shaded dark blue. The identified residues with chemical shift perturbations > 0.2 ppm (0.4 ppm maximum) are shaded in medium purple and the perturbations < 0.2 ppm are shaded in medium blue. The color scheme is based on palettes for color blindness and interpretability in grayscale [2].

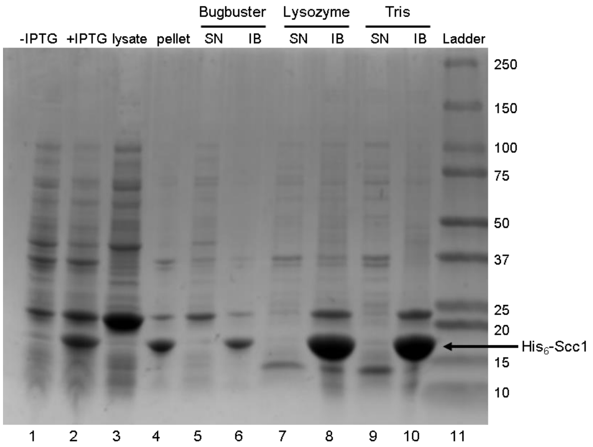

Figure S7. SDS-PAGE analysis of the His6-Scc1 inclusion body purification. Lanes are numbered at the bottom. Lanes 1 and 2 are proteins in the culture before and after induction with IPTG. Lanes 3 and 4 are the clarified lysate and the pellet after cell lysis. Lanes 5 and 6 are the inclusion body and supernatant from washing the pellet in Lane 4 with 1X BugBuster in Tris buffer. Lanes 7 and 8 are the inclusion body and supernatant from the second wash step with lysozyme in Tris buffer. Lanes 9 and 10 are the inclusion body and supernatant from the last wash step with Tris buffer. Lane 11 is the Bio-Rad Precision Plus protein ladder with molecular weights listed in kDa to the right. The His6-Scc1 (18.8 kDa) band is indicated with an arrow.

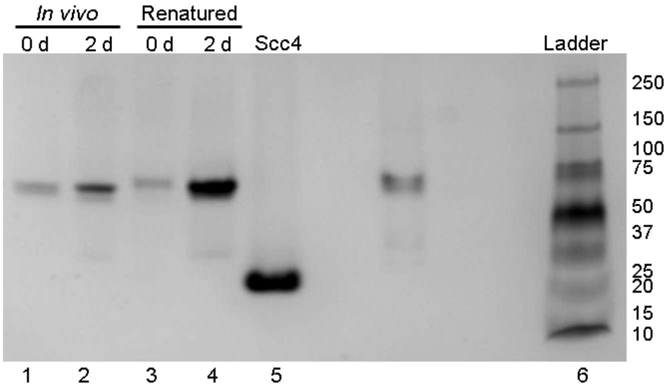

Figure S8. Full gel image of the native gel from Figure 6A. Lane numbers across the bottom correspond to the lane numbers in Figure 6A.

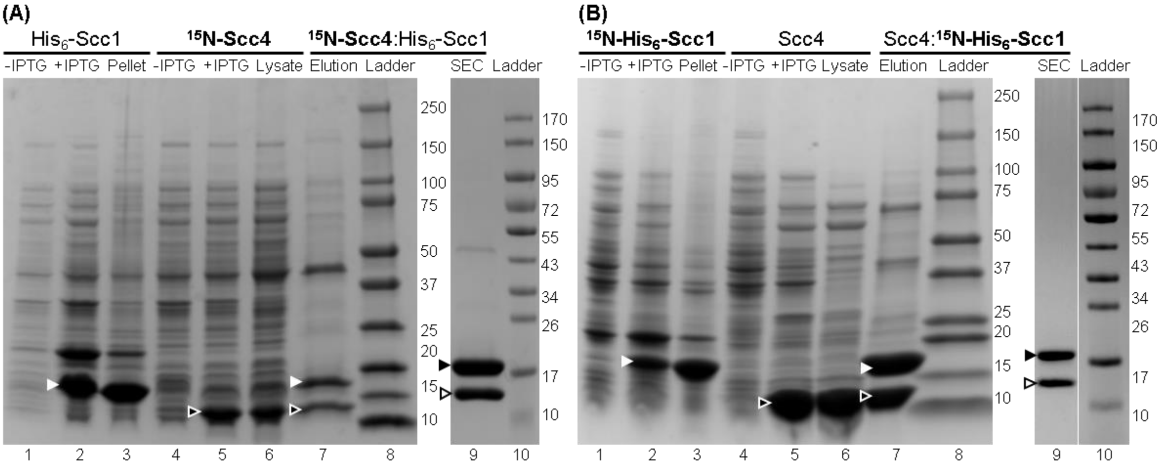

Figure S9. SDS-PAGE analysis of the renatured and chain-selectively labeled Scc4:His$_6$-Scc1 complexes: (A) ^{15}N-Scc4:His$_6$-Scc1 and (B) Scc4:^{15}N-His$_6$-Scc1. Filled arrow heads indicate the His$_6$-Scc1 bands and open arrow heads indicate the Scc4 bands. The bold font indicates the protein that is ^{15}N-labeled. (A) Samples of the cultures before and after induction with IPTG and expressing His$_6$-Scc1 (lanes 1 and 2) or ^{15}N-Scc4 (lanes 4 and 5) are shown. The His$_6$-Scc1 pellet (lane 3) and ^{15}N-Scc4 clarified lysate (lane 6) were used to make the renatured complex. Lanes 7 and 9 show the purification of the renatured complex by Ni-IMAC and SEC, respectively. Lane 8 is the Bio-Rad Precision Plus protein ladder and lane 10 is the Fisher BioReagents EZ-Run Rec pre-stained protein ladder with molecular weights listed to the right of each lane in kDa. (B) The same samples with opposite isotopic labeling are shown with the same lanes numbers as in (A). The difference between (B) and (A) is the labeling of the proteins with ^{15}N-His$_6$-Scc1 and unlabeled Scc4.

```
13 d1xkpc1 Probab=97.69   E-value=0.001   Score=41.63   Aligned_columns=112   Identities=19%

Q ss_pred               HHHHHCCCCCEEECCCCEEEEEECCEEEEEEEECCCCEEEEEECCCCC-CCCCCHHHHHH
Q ss_conf               9998298765047889819999889489999976864266430146 78-97546689999
Q Scc4-20190820      9 FVAYMGVASELEFDADGSYVFPISSLVRMRVRQNADEEIIISAFLGEI-PASMDIEKAYA   67 (133)
Q Consensus          9 f~~~lgl~~~L~~D~~g~C~L~id~~~~i~l~~~~d~~l~i~a~lg~l-P~~~~~~~~~~~   67 (133)
                        +++.+|-|  .-.|. |.+++.|| ...+-..++++-.+-.....++ .++....+++|
T Consensus          6 LAa~LGqP--FvADq-G~YhLrID-~~v~L~p~g~eLlL~tPl~~~~l~d~~~~~~~LLr   61 (121)
T d1xkpc1          6 LAASLGRP--FVADQ-GVYRLTID-HLVMLAPHGSELVLRTPIDAPMLREGNNVNVTLLR   61 (121)
T ss_dssp              HHHHTTCC--CCCCC-CCEEEECC-CEEEEEEETTEEEEEEECCCGGGEETTEECHHHHH
T ss_pred              HHHHCCCC--EECCC-CEEEEEEE-EEEEEEECCCEEEEECCCCCCCCCCCCCCCHHHHH
T ss_conf              78651883--00027-74889821-067886059926773233311124612223899 99


Q ss_pred               HHHHHHCCCCCCCCEEEEECCCCCEEEEEEEECCC-CCHHHHHHHHHHHHHHHHHHHHHH
Q ss_conf               99975145766654289981799689998433 44-69999999999999999999986
Q Scc4-20190820     68 RMMEGNLFGQETGGAALGLDSDGHAVLVRRVPGE-VSQEDFASYIESVLNYAEAWLEDL  125 (133)
Q Consensus         68 ~LL~~N~~~~~t~g~~lgl~~~~~~~vl~~~~~~a~-l~~~~f~~~l~~Fv~~a~~W~~~l  125 (133)
                        +||+...-|.....-++-+|..+++++..++--+ ++.+-+++.+.+ +...++..-+|
T Consensus         62 ~Llqqv~~W~rryPqalVLDd~G~L~LeARL~L~~LD~~~L~e~l~Q-iaLLE~L~PqL  119 (121)
T d1xkpc1         62 SLMQQALAWAKRYPQTLVLDDCGQLVLEARLRLQELDTHGLQEVINQ-LALLEHLIPQL  119 (121)
T ss_dssp              HHHHHHHHHTTTCCCEEEECTTSCEEEEEEEEGGGCCHHHHHHHHCC-HHHHHHHGGGG
T ss_pred              HHHHHHHHHHHHCCCEEEEECCCCCHHHHHHHHCCCCHHHHHHHHHH-HHHHHHHHHCC
T ss_conf              9999999875528914887023300554223011149899999999 9-78698760315
```

Figure S10. Phyre2 [3] alignment of *CT* Scc4 and *Yersinia pestis* YscB from PDB 1XKP, chain C [4]. Details for interpreting the alignment are available on the Phyre2 web page [5].

```
12 d1xkpb1 Probab=97.60   E-value=0.0038   Score=37.47   Aligned_columns=113   Identities=17%

Q ss_pred                HHHHHHHHHHHCCCC-EEECCCCEEEEEECCCEEEEEEEEECCCCCEEEEEEEECCCCCCCC
Q ss_conf                999999999860992-55768982799985985899999859987599992026789744
Q CtSccl-intensi    5 FEQLLTELGTQINSP-LTPDSNNACIVRFGYNNVAVQIEEDGNSGFLVAGVMLGKLPENT   63 (146)
Q Consensus         5 fe~lL~elg~~lgl~-L~~D~~g~C~L~~dd~~l~i~l~~~~~~~~~~L~l~~~l~~lp~~~   63 (146)
                       ++..++||+|.+|++ +.+-+ +.-.|-|++-+ +++||...+.=.+++-.++.+.
T Consensus         3 i~~~v~eF~r~mG~~~~~~~~~-~~v~L~~e~~G-tL~iE~~~e~L~l~Lar~l~~~----   56 (119)
T d1xkpb1           3 IEPIISHFCQDLGVPTSSPLS-PLIQLEMAQSG-TLQLEQHGATLTLWLARSLAWH----   56 (119)
T ss_dssp               THHHHHHHHHHTTCCCCSSCC-SEEEEEETTTE-EEEEEEETTEEEEEEEEEECCGG----
T ss_pred               HHHHHHHHHHHCCCCCCCCCC-CEEEEEECCCC-EEEEEECCCEEEEEEEECCCCCC----
T ss_conf               023699998755996534566-42699984762-4999624887999982338821----


Q ss_pred                CCHHHHHHHHHHHCCCCCCCCEEEEEECCCCCEEEEEEEECHHHCCHHHHHHHHHHHHHHH
Q ss_conf                668999999996345512676189983689609999962444489999999999999999
Q CtSccl-intensi   64 FRQKIFKAALSINGSPQSNIKGTLGYGEISNQLYLCDRLNMTYLNGEKLARYLVLFSQHA  123 (146)
Q Consensus        64 ~r~~~~~~~LL~~Nl~~~~t~g~~lgl~~~~~~iiL~~~~~~~~~~l~~~~l~~~L~~Fv~~a  123 (146)
                        +.+..+++|+.-...+..---.=|+. ..+++++..+++...++...+.+++...+..-
T Consensus        57 -~~~~~~~kaL~l~hy~~~~~pl~aG~~-ge~~Lvl~~~l~~~~~tl~~le~A~~~L~~l~  114 (119)
T d1xkpb1          57 -RCEDAMVALTLTAAQSGALPLRAGWL-GESQLVLFVSLDERSLTLPLLHQAFEQLLRLQ  114 (119)
T ss_dssp               -GHHHHHCCTTTTSCCCCSSCCEEEEE-TTTEEEEEEEEEEGGGCCHHHHHHHHHHHHHHH
T ss_pred               -CHHHHHHHHHHHCCCCCCCCEEEEEC-CCCEEEEEEEEEECHHHCCHHHHHHHHHHHHHHH
T ss_conf               -20669999986377778742010026-99819999981844336799999999999999


Q ss_pred                HH
Q ss_conf                99
Q CtSccl-intensi  124 NI  125 (146)
Q Consensus       124 ~~  125 (146)
                       +.
T Consensus       115 qe  116 (119)
T d1xkpb1         115 QE  116 (119)
T ss_dssp               HH
T ss_pred               HH
T ss_conf               98
```

Figure S11. Phyre2 [3] alignment of *CT* Scc1 and *Yersinia pestis* SycN from PDB 1XKP, chain B [4]. Details for interpreting the alignment are available on the Phyre2 web page [5].

```
3 c1xkpA_ Probab=100.00  E-value=9.4e-40  Score=268.46  Aligned_columns=212  Identities=14%

Q ss_pred                 CCCCCCCCCCCHHHHHHHHHHHHHHHHHHHHHHHHHHHHHHHHHHHHHHHHHHCCCCHH
Q ss_conf                 355676300063654201356678876755778888554445689888876431022136
Q CtCopN-intensi   37 QEASEASMLKECEDLINPAAATRIKKKEEKFESLEARRKPTADKAEKKSESTEEKGDTPL   96 (421)
Q Consensus        37 ~~~s~ls~~~~~~~el~~~Aa~~r~~~~eek~~~~~~~r~~~~a~~~E~~~~~~~~k~~~dl   96 (421)
                          ++|.+++...++......|+++-..--..+          ..+...|+++     +....
T Consensus         2 ~~Ge~v~~~~~~~~s~ladaaEEmS~l~~~~----------~~k~~~r~s~-----~~~~~   46 (217)
T c1xkpA_           2 FRGESVQIVSGTLQSIADMAEEVTELSLDR----------LSDSQARVSD-----VEEQV   46 (217)
T ss_dssp                BTTBCCCCCCSSCCCHHHHHHHHCCCCCCC----------CCCHHHHHHH-----HHHHH
T ss_pred                CCCCCEEECCCCCCHHHHHHHHHHHHHHHH----------HHHHHHHHHH-----HHHHH
T ss_conf                768700551675662876999999999998----------7876643335-----66799


Q ss_pred                 HHHHHCCCCCCCHHHHHHHHHHHCCCCCHHHHHHHHHHHCCHHHHHHHHHHHHHHHCCCC
Q ss_conf                 787301111111588999997406799989999999987488126789999999622463
Q CtCopN-intensi   97 EDRFTEDLSEVSGEDFRGLKNSFDDDSSSDEILDALTSKFSDPTIKDLALDYLIQIAPSD  156 (421)
Q Consensus        97 ~dr~~~~~~~e~~~q~L~~~R~al~~d~S~~~il~~~~e~f~DpS~q~~AL~~l~q~~~~~  156 (421)
                          .+..++.+.+-  +++..+-..+..+++..++++.++++|+|||++|++|.++.+....+
T Consensus        47 ~~~~~~e~~d~--~k~~~l~~~~~~~~~~~~~~~l~~a~q~FpD~Sd~~laL~~~~~~~~~~~~  104 (217)
T c1xkpA_          47 NQYLSVPELEQ--QNVSELLSLLSNSPNISLSQLAYLEGSEEPSEQFMLCGLRDALGRPE  104 (217)
T ss_dssp                HHHHCCCCCCC--CHHHHHHHHHSSCSSCCTTTCCTTTCCCCHHHHCCHHHHHHHCCCGG
T ss_pred                HHHHHHHHHHH--HHHHHHHHHHCCCCCHHHHHHHHHHHCCCCHHHHHHHHHHHHHCCCC
T ss_conf                99974234689--999999999424689599999999758990289999999997046 89


Q ss_pred                 H-HHHHHHHHHHHHHHHHCCCHHHHHHHHHHHHHHHHHHHHCCHHHHHHHHHHHHHCCCCH
Q ss_conf                 2-5899999999999720657779888899999999986504898999999998638622
Q CtCopN-intensi  157 G-KLKSALIQAKHQLMSQNPQAIVGGRNVLLASETFASRANTSPSSLRSLYFQVTSSPSN  215 (421)
Q Consensus       157 g-el~~~l~~a~~el~~~~g~aIrAGiNvAl~A~~Fa~~l~~sp~~LRdlYR~~v~~~s~  215 (421)
                          . ++..++.++++++++++++|++|+||+|||++|+.|+++++++++|+.||++||+||.....
T Consensus       105 ~~~~~~li~~a~~~l~~~~~~~~i~AGiNvAl~A~~Fs~~~~~~~~~~~LR~lYR~~v~~~~~~  164 (217)
T c1xkpA_         105 LAHLSHLVEQALVSMAEEQGETIVLGARITPEAYRESQSGVNPLQPLRDTYRDAVMGYQG  164 (217)
T ss_dssp                GHHHHHHHHHHHHHHHHHHHHHHHHHHHHHHHHHHHHHHHHTTSSCCHHHHHHHHHCCCC
T ss_pred                HHHHHHHHHHHHHHHHHCCHHHHHHHHHHHHHHHHHHHHCCCCHHHHHHHHHHHHCCCCC
T ss_conf                788999999999999820737779865146899999998741689899999999617654


Q ss_pred                 HHHHHHHHHHCCHHHHHHHHHHHHHHHHHHHHHCCCCCCHHHHHHHHHHHHH
Q ss_conf                 7999999984690459999999999999855542188889999999987998
Q CtCopN-intensi  216 CANLHQMLASYSPSEKTAVMEFLVNGMVADLKSEGPSIPPAKLQVYMTELSN  267 (421)
Q Consensus       216 ~d~y~s~L~rYg~~~~~~~~~~vi~FL~~aL~~DL~S~~PS~~~~~~~L~~llt~l~~  267 (421)
                          +..+++||++||+++|++|++|| +||++||+|++||||+ +|+++|++|.+
T Consensus       165 ~~~~~~~~~i~~fg~~~~~~~~vl~fl-~aL~~Dl~S~~PS~~~-el~~lm~~l~k  214 (217)
T c1xkpA_         165 IYAIWSDLQRFPNGDIDSVILFL-QALSADLQSQQSGSGR-ELGIVISDLQL  214 (217)
T ss_dssp                HHHHHHHHCCCTTSCHHHHHHHH-CCHHHHHHHCCCSTTT-CCHHHHHHHCC
T ss_pred                HHHHHHHHHHCCHHHHHHHHHHH-HHHHHHHHHHCCCCHH-HHHHHHHHHHH
T ss_conf                89999999823925599999999-9999766423899736-99999999866
```

Figure S12. Phyre2 [3] alignment of *CT* CopN and *Yersinia pestis* YopN from PDB 1XKP, chain A [4]. Details for interpreting the alignment are available on the Phyre2 web page [5].

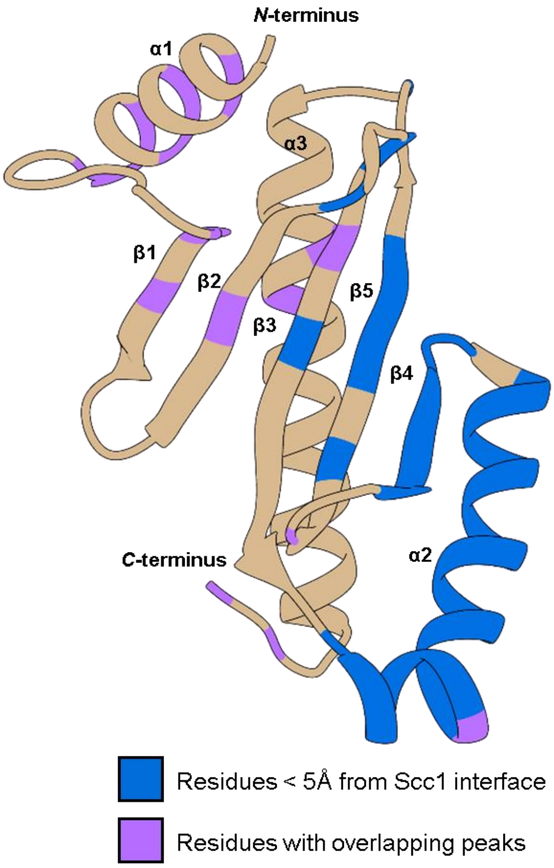

Figure S13. Scc4 homology model with Scc1 interface and overlapping resonances. Scc4 residues less than 5 Å from the homology model-predicted Scc1 interface are shown in blue. Scc4 residues with $^{1}H,^{15}N$-HSQC peaks that overlap peaks from the $^{1}H,^{15}N$-HSQC spectrum of ^{15}N-Scc4:His$_6$-Scc1 are shown in purple.

References

1. Ukwaththage, T.O.; Tonelli, M.; Macnaughtan, M.A. Backbone and sidechain resonance assignments and secondary structure of Scc4 from *Chlamydia trachomatis*. *Biomol NMR Assign* **2020**, *14*, 301-307, 10.1007/s12104-020-09965-4.
2. Designing for color blindness: color choices and transformations for deuteranopia and other afflictions. Available online: http://mkweb.bcgsc.ca/colorblind/index.mhtml.
3. Kelley, L.A.; Mezulis, S.; Yates, C.M.; Wass, M.N.; Sternberg, M.J.E. The Phyre2 web portal for protein modeling, prediction and analysis. *Nat Protoc* **2015**, *10*, 845-858, 10.1038/nprot.2015.053.
4. Schubot, F.D.; Jackson, M.W.; Penrose, K.J.; Cherry, S.; Tropea, J.E.; Plano, G.V.; Waugh, D.S. Three-dimensional structure of a macromolecular assembly that regulates type III secretion in *Yersinia pestis*. *J Mol Biol* **2005**, *346*, 1147-1161, 10.1016/j.jmb.2004.12.036.
5. Available online: http://www.sbg.bio.ic.ac.uk/phyre2/html/help.cgi?id=help/interpret_normal#alignment.
